# Genomic Characterization Provides New Insights Into the Biosynthesis of the Secondary Metabolite Huperzine a in the Endophyte *Colletotrichum gloeosporioides* Cg01

**DOI:** 10.3389/fmicb.2018.03237

**Published:** 2019-01-08

**Authors:** Xincong Kang, Chichuan Liu, Pengyuan Shen, Liqin Hu, Runmao Lin, Jian Ling, Xingyao Xiong, Bingyan Xie, Dongbo Liu

**Affiliations:** ^1^Horticulture and Landscape College, Hunan Agricultural University, Changsha, China; ^2^Hunan Provincial Key Laboratory of Crop Germplasm Innovation and Utilization, Hunan Agricultural University, Changsha, China; ^3^State Key Laboratory of Subhealth Intervention Technology, Changsha, China; ^4^Institutes of Vegetables and Flowers, Chinese Academy of Agricultural Sciences, Beijing, China; ^5^Hunan Co-Innovation Center for Utilization of Botanical Functional Ingredients, Changsha, China

**Keywords:** endophyte, genome, differentially expressed genes, biosynthesis, histone modification, gene knockout

## Abstract

A reliable source of Huperzine A (HupA) meets an urgent need due to its wide use in Alzheimer's disease treatment. In this study, we sequenced and characterized the whole genomes of two HupA-producing endophytes, *Penicillium polonicum* hy4 and *Colletotrichum gloeosporioides* Cg01, to clarify the mechanism of HupA biosynthesis. The whole genomes of hy4 and Cg01 were 33.92 and 55.77 Mb, respectively. We compared the differentially expressed genes (DEGs) between the induced group (with added extracts of *Huperzia serrata*) and a control group. We focused on DEGs with similar expression patterns in hy4 and Cg01. The DEGs identified in GO (Gene ontology) and KEGG (Kyoto Encyclopedia of Genes and Genomes) pathways were primarily located in carbon and nitrogen metabolism and nucleolus, ribosome, and rRNA processing. Furthermore, we analyzed the gene expression for HupA biosynthesis genes proposed in plants, which include lysine decarboxylase (LDC), copper amine oxidase (CAO), polyketides synthases (PKS), etc. Two LDCs, one CAO, and three PKSs in Cg01 were selected as prime candidates for further validation. We found that single candidate biosynthesis-gene knock-out did not influence the HupA production, while both LDC gene knock-out led to increased HupA production. These results reveal that HupA biosynthesis in endophytes might differ from that proposed in plants, and imply that the HupA-biosynthesis genes in endophytic fungi might co-evolve with the plant machinery rather than being acquired through horizontal gene transfer (HGT). Moreover, we analyzed the function of the differentially expressed epigenetic modification genes. HupA production of the histone acetyltransferase (HAT) deletion mutant Δ*CgSAS-2* was not changed, while that of the histone methyltransferase (HMT) and histone deacetylase (HDAC) deletion mutants Δ*CgClr4*, Δ*CgClr3*, and Δ*CgSir2-6* was reduced. Recovery of HupA-biosynthetic ability can be achieved by retro-complementation, demonstrating that HMT and HDACs associated with histone modification are involved in the regulation of HupA biosynthesis in endophytic fungi. This is the first report on epigenetic modification in high value secondary metabolite- producing endophytes. These findings shed new light on HupA biosynthesis and regulation in HupA-producing endophytes and are crucial for industrial production of HupA from fungi.

## Introduction

**Figure 1:**
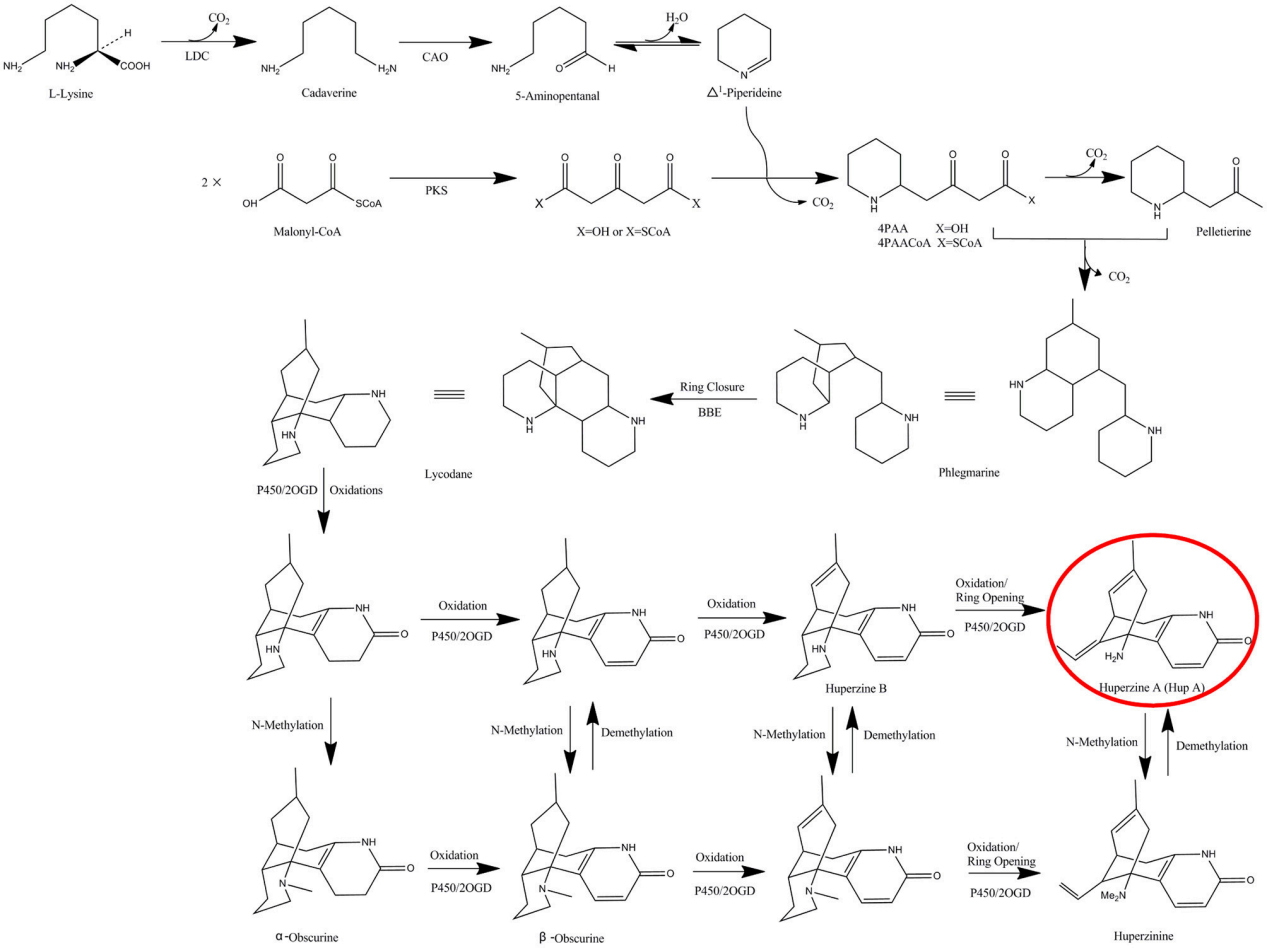
The proposed biosynthetic pathway of HupA in *H. serrata*. LDC, lysine decarboxylase; CAO, copper amine oxidase; PKS, polyketides synthases; BBE, berberine bridge enzyme; 2OGD, 2-oxoglutarate-dependent dioxygenases.

(-)-Huperzine A (HupA, [(5R, 9R, 11E)-5-amino-11-ethylidene-5, 6, 9, 10-tetrahydro-7-methyl-5, 9-methano-cycloocteno [b] pyridine-2(1H)-one], Figure 1), is a competitive and reversible inhibitor of acetyl cholinesterase that is known to improve general cognitive function, global status, behavioral disturbance, and functional performance (Ha et al., 2011). It is found and extracted from a small group of the plant family Huperziaceae with a very low yield (0.0047 to 0.025% in *Huperzia serrata*) (Ma et al., 2005; Ha et al., 2011). These plants are not abundant and are only found in very specialized habitats (Ma et al., 2005). Tissue culture of the host plants is rarely successful, and the rigid molecular configuration of HupA makes chemical synthesis difficult (Ha et al., 2011). A reliable and sustainable source is required due to the high demand for HupA in Alzheimer's disease treatment.

After the discovery of the Taxol-producing endophytic fungus, endophytes have been the subject of increasing focus for many high-value bioactive secondary metabolites (SMs) (Venugopalan and Srivastava, 2015). Their host plants have often become endangered or endemic due to excessive exploitation (Venugopalan and Srivastava, 2015). Microbial fermentation has certain inherent advantages, such as simple and inexpensive culture, fast growth, ease of yield enhancement and scaling up, simple genetics, and ease of manipulation (Venugopalan and Srivastava, 2015). Several HupA-producing endophytes, such as *Shiraia* sp. Slf14, *Colletotrichum gloeosporioides* ES026, *Trichoderma sp*., and *Paecilomyces tenuis* YS-13, have been isolated from *H. serrata* (Zhu et al., 2010; Wang et al., 2011; Zhao et al., 2013; Dong et al., 2014; Su and Yang, 2015). However, few reports explore the mechanism of the production of host plant associated SMs in endophytes, instead screening the SM-producing strains (Venugopalan and Srivastava, 2015).

The biosynthetic pathway of HupA was proposed by Ma et al. in the Lycopodiaceae family, according to the chemical synthesis of lycopodium alkaloids (Figure 1) (Ma and Gang, 2004). The proposed pathway starts with the decarboxylation of lysine (by lysine decarboxylase, LDC), which is then transformed to Δ1-piperideine via 5-amiopentanal (by copper amine oxidase, CAO). At the same time, two malonyl-CoA are condensed to form acetonedicarboxylic acid (perhaps by polyketide synthase, PKS). Δ1-piperideine is then coupled to acetonedicarboxylic acid (or its bisCoA ester) to form 4-(2-piperidyl) acetoacetate (4PAA) (or 4-(2- piperidyl) acetoacetyl-CoA, 4PAACoA). 4PAA/4PAACoA is then decarboxylated (4PAACoA is perhaps hydrolyzed first) to form pelletierine. Pelletierine and 4PAA/4PAACoA, or some derivatives, are coupled, accompanied by requisite decarboxylation, to form phlegnarine. After oxidative ring closure of phlegnarine to form lycodane (likely by berberine bridge enzyme, BBE), other oxidative modifications lead a series of precursors to HupA, presumably catalyzed by cytochrome P450s or 2-oxoglutarate-dependent dioxygenases (2OGD) (Ma and Gang, 2004). However, among these putative HupA biosynthetic genes, only LDC, CAO, and PKS genes were cloned, characterized and expressed *in vitro* (Sun et al., 2012; Yang et al., 2016; Xu et al., 2017). None of these putative biosynthetic genes was confirmed by *in vivo* experiments to establish a direct relationship with HupA. It has long been existing of the conflicts about the source of the endophytic biosynthetic genes (Venugopalan and Srivastava, 2015). Fungal genes showed homology to the plant counterparts, supporting the horizontal gene transfer (HGT) theory (Staniek et al., 2009; Zhang et al., 2009), while low similarity was found in fungi with the corresponding plant genes, supporting co-evolution rather than HGT (Hedden et al., 2001; Yang et al., 2014). Up to now, there has been no systematic analysis of the HupA biosynthesis mechanism in endophytic fungi, nor of the origin.

In most cases, the genes coding for SM-biosynthesis enzymes are clustered in fungal genomes and silenced by heterochromatic histone marks (Keller and Hohn, 1997). Typical heterochromatic marks are characterized by both hypoacetylation of the histone tails and methylation of histone H3 on lysine 9 or lysine 27 (Bannister et al., 2001; Nakayama et al., 2001; Shahbazian and Grunstein, 2007; Pettit, 2011). The “closed” heterochromatic structures could be reversed during SM activation. In filamentous fungi, chromatin structure and function has only been studied in a few model systems, such as *Neurospora crassa, Aspergillus nidulans*, and *Penicillium chrysogenum* (Strauss and Reyes-Dominguez, 2011). The first evidence of chromatin involvement in SM regulation was that HDAC deletion leads to transcriptional activation of two telomere-proximal SM gene clusters and to an elevated level of sterigmatocystin and penicillin in *A. nidulans* (Shwab et al., 2007). Subsequently, it was found that during the active growth phase, the silent sterigmatocystin gene cluster is marked by H3K9me3 and contains high levels of the heterochromatin protein-1 (HepA) (Reyes-Dominguez et al., 2010). Upon growth arrest and activation of SM, HepA and trimethylated H3K9 levels decrease concomitantly with increasing levels of acetylated histone H3 (Reyes-Dominguez et al., 2010). However, there has yet been no report on the epigenetic regulation of SM production in endophytes.

In this study, we sequenced the whole genomes of two HupA-producing endophytes (*Penicillium polonicum* hy4 and *Colletotrichum gloeosporioides* Cg01) from *Huperzia serrata* and compared the differentially expressed genes (DEGs) between the induced group (with added extracts of *H. serrata*) and a control group. We characterized the whole genomes and obtained the KEGG (Kyoto Encyclopedia of Genes and Genomes) enrichment pathways and GO (Gene Ontology) enrichment function clusters of these DEGs. The focus of this study is the genes proposed in plants to biosynthesize HupA, which include LDC, CAO, PKS, BBE, P450, and 2OGD genes, as well as the epigenetic modification genes. It is the first report on epigenetic modification analysis in high value SM-producing endophytic fungi. This study sheds new light on HupA biosynthesis and regulation in HupA-producing endophytic fungi and is the basis of further synthetic biology study for industrial production of HupA from fungi.

## Materials and Methods

### Fungal Strains

The endophytic fungi *P. polonicum* hy4 and *C. gloeosporioides* Cg01 that produces HupA were isolated and screened from *H. serrata*, which collected from Nanping, Fujian, China. Two strains were purified by single-spore isolation and identified by ITS sequencing. The strains were cultured on Potato Dextrose Agar medium at 28°C and deposited at the China Center for Type Culture Collection (hy4: CCTCC No. M2010086, Cg01: CCTCC No. AF2018024; Wuhan, China).

**Figure 2:**
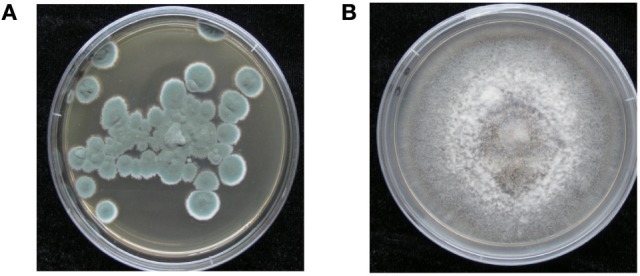
Morphology of *P. polonicum* hy4 **(A)** and *C. gloeosporioides* Cg01 **(B)** on potato dextrose agar.

Colony of *P. polonicum* hy4 on PDA was cyan, wrinkled, good sporulation, margin slightly irregular, reverse light brown (Figure 2). Colony of *C. gloeosporioides* Cg01 grown from single conidia on PDA about 8 cm diam after 5 d, aerial mycelium sparse, white, or gray-white, surface of colony with numerous acervuli, some with dark bases, with orange conidial ooze (Figure 2).

### Extraction of Genomic DNA and Total RNA for Sequencing

*P. polonicum* hy4 and *C. gloeosporioides* Cg01 were cultured in potato dextrose broth (PDB) at 28°C for 3–5 days. Mycelia were collected by filtering the liquid culture and used for genomic DNA extraction by a modified CTAB method as previously described (Kang et al., 2011).

Total RNA was extracted from different samples of *P. polonicum* hy4 and *C. gloeosporioides* Cg01 using TRIzol (Takara, Dalian, China). Control group: either hy4 or Cg01 cultured in PDB enriched media (with added peptone 2g/L, yeast extract powder 2 g/L, KH_2_PO_4_ 1.5 g/L, MgSO_4_ 0.5 g/L, NaCl 0.3 g/L in PDB, pH 6.5) for 12 days at 28°C; induced group: either hy4 or Cg01 cultured in PDB enriched media for 5 d at 28°C, extracts of *H. serrata* were added, and subsequently cultured another 7 d. *P. polonicum* hy4 only produced HupA with the extract of *H. serrata* (535.54 μg/L), while *C. gloeosporioides* could produce HupA in small amounts (21.04 μg/L) alone and increased to 556.21 μg/L with the extract of *H. serrata* (Figure S1).

RNA purity and integrity were verified by electrophoresis on 1% agarose gel. RNA integrity was assessed using the RNA Nano 6000 Assay Kit of the Bioanalyzer 2100 system (Agilent Technologies, CA, USA). RNA quantity was measured using Qubit RNA Assay Kit in Qubit 2.0 Flurometer (Life Technologies, CA, USA). The RNA samples were sent to Novogene (Beijing, China) for RNA sequencing.

### Sequencing and Assembly

The genomes of *P. polonicum* hy4 and *C. gloeosporioides* Cg01 were sequenced with Illumina HiSeq 2000, using a whole-genome shotgun-sequencing strategy. Three sequencing libraries were constructed for *P. polonicum* hy4, with insert sizes of 170 bp, 750 bp, and 5 kb. Four libraries were for *C. gloeosporioides* Cg01, with insert sizes of 170 bp, 300 bp, 500 bp, and 5 kb. Clean reads were assembled using ALLPATHS-LG to construct scaffolds (Gnerre et al., 2011). The Whole Genome Shotgun projects have been deposited at DDBJ/ENA/GenBank under the accession QPIC00000000 (*P. polonicum* hy4) and QRFY00000000 (*C. gloeosporioides* Cg01). The versions described in this paper are version QPIC01000000 and QRFY01000000, respectively.

The RNA sequencing was performed with an Illumina HiSeq 4000 and 125 bp paired-end reads were generated. Clean reads were obtained by removing reads containing adapter, reads containing ploy-N and low quality reads from raw data. TopHat v2.0.12 was used to align the clean reads to the reference genomes (Trapnell et al., 2009). Cufflinks (Trapnell et al., 2010) was then used to calculate the expected number of fragments per kilobase of transcript sequence per millions base pairs sequenced (FPKM).

### Gene Annotation and Phylogenetic Analysis

To increase the accuracy of gene prediction, GeneMark-ES v4.32 and AUGUSTUS v2.7 were used to obtain *ab initio* prediction of gene structure (Stanke and Morgenstern, 2005; Ter-Hovhannisyan et al., 2008). The data were combined by Maker v2.31.8 to produce a consensus gene set (Holt and Yandell, 2011). For functional annotation, protein sequences were blasted against a series of protein databases, including NCBI RefSeq (National Center of Biotechnology Information Reference Sequence), UniProtKB (UniProt Knowledgebase) Swiss-Prot, KEGG, KOG (EuKaryotic Orthologous Groups), GO, and Pfam. PKSs were predicted by SMURF (Secondary Metabolite Unique Regions Finder, http://www.jcvi.org/smurf) and antiSMASH (antibiotics and Secondary Metabolite Analysis Shell, https://fungismash.secondarymetabolites.org/). Phylogenetic trees for PKS and CAO were constructed using the maximum-likelihood approach implemented in MEGA v6.06 (Tamura et al., 2013). For LDC, CAO, and BBE gene prediction, we downloaded all LDC, CAO, and BBE genes in NCBI and conducted whole-genome Blastp. The Fungal Cytochrome P450 database (http://drnelson.uthsc.edu/cytochromeP450.html) was used to identify P450 by Blastp, with an e-value cutoff of 1e-50. All proteins containing one 2OG-FeII_Oxy motif (Pfam ID: PF03171) were regarded as 2OGD (Kawai et al., 2014). Orthologous genes between hy4 and Cg01 were identified by OrthoMCL (Li et al., 2003).

### Differentially Expressed Gene Analysis

Differential expression analysis of two groups (three biological replicates per group) was performed using Cufflinks (Trapnell et al., 2010). Abundances are reported as normalized FPKM. Cufflinks determines differential expression in digital gene expression data using a model based on the negative binomial distribution. The resulting *P*-values were adjusted using the Benjamini-Hochberg correction for controlling the false discovery rate. Genes with a *Q*-value (adjusted *P*-value) ≤ 0.05 were considered differentially expressed. Genes with a *Q*-value ≤ 0.05 and log_2_(fold_change) | ≥ 1 were considered significantly differentially expressed and used for volcano plotting and KEGG and GO enrichment analysis.

### Gene-Knockout and Retro-Complementation

The target genes were deleted by the split-marker homologous recombination knockout method (Liang et al., 2014). The method uses gene-knockout cassettes containing overlapping truncations of selectable marker to integrate homologs in transformation (Liang et al., 2014). In this study, the marker gene was the hygromycin-resistance gene, allowing selection of the deleted mutation on selective media. For gene retro-complementation, the plasmid pKOV21 was used as the vector and G418 was used as the selected marker. Fragments with HMT (histone methyltransferase) or HDAC (histone deacetylase) genes, including the native promoter and terminator, underwent PCR and were cloned into the vector pKOV21 with the In-fusion HD Cloning Kit (Takara). The deletion and retro-complementation primers used are listed in Table S1. Retro-complementation vectors, containing the retro-complementation fragments, were confirmed by sequencing. Protoplasts were produced by 20 g/L Driselase (Sigma-Aldrich, St. Louis, MO, USA) and lywallzyme (Guangdong Institute of Microbiology, Guangzhou, China) digestion for 4–5 h at 33°C. Gene-knockout cassettes and retro-complementation vectors were transformed by the polyethylene glycol-mediated protoplast transformation method (Liang et al., 2014). After incubation at 28°C for 3–5 d, transformed colonies were transferred to PDA plates with 200 μg/mL hygromycin B or 500 μg/mL G418 sulfate for the second round of selection. The deletion mutants were verified by genomic DNA extraction and PCR identification with D-F/D-R, 1F/2R, and 5F/4R, and the retro-complementation strains were verified by PCR with primers of D-F/D-R (Table S1).

### High Performance Liquid Chromatography (HPLC) Analysis of HupA

Strains were cultured as described above, and three replicates of culture media with only extracts of *H. serrata* were also prepared. The cultured media after filtration were concentrated on a rotary evaporator and then extracted twice with chloroform. The combined chloroform extracts were evaporated to dryness. The dry residues were dissolved with 2 mL methanol and filtered through 0.22-μm polytetrafluoroethylene syringe filters before HPLC analysis. A C18 column (Agilent Eclipse plus-C18, 5 μm, 4.6 × 250 mm, Agilent Technologies, Santa Clara, CA, USA) was used for separation at 30°C. The mobile phases consisted of ammonium acetate (15 mmol/L) and methanol (70: 30, vol/vol, pH 6.0) at a flow rate of 1 mL/min. Detection of HupA was performed at a wavelength of 308 nm. HupA produced by the mutant was calculated as the total HupA content in the mutant fermentation broth minus HupA content of the broth with only extract of *H. serrata*.

## Results

### Genome Sequencing, Assembly, and Characterization

**Figure 3:**
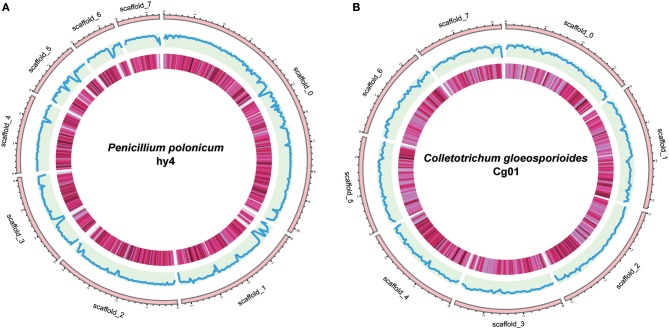
Genome organization and gene distribution of *P. polonicum* hy4 **(A)** and *C***.** *gloeosporioides* Cg01 **(B)**. From the outside inward, the first circle represents the scaffolds. Scaffolds 0–7 represent the longest eight scaffolds of hy4 and Cg01. The second circle indicates the GC content with the ratio of GC sites per 50 kb (hy4: 0.22–0.54; Cg01: 0.37–0.59). The third circle indicates the gene density with the frequency of sites per 50 kb (deeper color indicates larger numbers; hy4: 0–28, Cg01: 2–26).

The assembled genome of hy4 was 33.92 Mb, and the total length of the eight largest scaffolds was 29.28 Mb (approximately 86.32% of the assembled genome) (Table 1, Figure 3). The longest scaffold of hy4 was ~10.0 Mb, and N50 was ~4.0 Mb (Table 1). The whole genome of Cg01 was assembled in 265 scaffolds, with a total length of 55.77 Mb (Table 1). The longest scaffold of Cg01 reached 3.5 Mb, with N50 of ~1.8 Mb (Table 1). The average GC content of hy4 was ~46.9%, and the ratio of GC sites per 50 kb was 22–54%, while those of Cg01 were much higher: ~53.2% and 37–59%, respectively (Figure 3).

**Table 1:** Genomic information for the two HupA-producing endophytes *P. polonicum* hy4 and *C. gloeosporioides* Cg01.

**Scaffold characteristics**	**hy4**	**Cg01**
Total number	32	265
scaffold num (≥1,000)	32	255
Total length (bp)	33,920,094	55,772,594
N50 (bp)	4,226,832	1,820,057
N90(bp)	1,168,368	518,011
Max length (bp)	10,177,925	3,505,042
Min length (bp)	1,373	968
**CONTIG CHARACTERISTICS**		
Total number	279	670
Contig num (≥1,000)	278	656
**GENOME CHARACTERISTICS**		
Genome assembly (Mb)	33.92	55.77
Whole GC content (%)	46.9	53.2
Number of protein-coding genes	11,696	17,713

A total of 11,696 and 17,713 protein-coding genes (PCGs) were predicted from the genomes of hy4 and Cg01, respectively. The lengths of PCGs in hy4 ranged from 156 to 19,539 bp, with an average size of 1,637 bp. There was a wider range of PCGs lengths in Cg01, from 156 to 31,747 bp. Genes were typically with exons, averaging 474 bp in hy4 and 504 bp in Cg01. The gene density measured as frequency of sites per 50 kb was 0–28 in hy4 and 2–26 in Cg01 (Figure 3).

**Figure 4:**
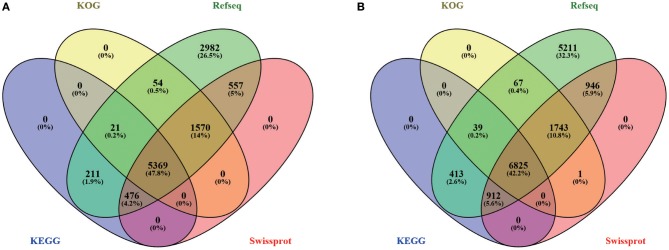
Venn diagram of gene annotation in four databases (KOG, Refseq, KEGG, and Swiss-Prot) of *P. polonicum* hy4 **(A)** and *C. gloeosporioides* Cg01 **(B)**.

To conduct functional annotation of the gene models, we used the Blastp search (*E*-value ≤ 1e-5) of the putative PCG sequences against several databases (KEGG, GO, KOG, Pfam, Swiss-Prot, and Refseq). In summary, 11,282 (96.46%) genes in hy4 and 16,218 (91.56%) genes in Cg01 have annotation information (Figure 4; Tables S2, S3), suggesting high quality of genome assembly and gene prediction. Among these annotated genes in hy4, 11,240 genes (99.6%) Blastp to Refseq showed an *e*-value ≤ 1e-5 and 10,393 genes (92.1%) had an *e*-value ≤ 1e-50. In Cg01, 16,156 (99.6%) genes had an *e*-value ≤ 1e-5 and 15,193 (93.7%) showed *e*-value ≤ 1e-50.

### RNA Sequencing, Identification and Functional Analysis of DEGs

About 4 Gb clean reads (removing sequencing adapters and low-quality bases) were generated for each sample (Table 2). Q20s of all samples were above 96.5% and Q30s were above 92% in both hy4 and Cg01 (Table 2). The GC content of each sample was almost 54% in hy4 and 57% in Cg01. Reads of all samples mapped ~90% of the reference genomes (Table 2). Among the mapped reads, ~91.90–93.60% mapped to the exons in hy4 samples, while ~75.80–79.70% mapped to the exons in Cg01 samples (Table 2).

**Table 2:** RNA sequencing information for the two HupA-producing endophytes *P. polonicum* hy4 and *C. gloeosporioides* Cg01 in the control group and induced group.

**Sample name**	**Clean bases (G)**	**Q20 (%)**	**Q30 (%)**	**GC content (%)**	**Total mapped (%)**	**Mapped to exons (%)**	**Mapped to introns (%)**	**Mapped to intergenic (%)**
hy4_a	4.26	96.98	92.55	53.97	90.18	92.70	0.40	6.90
hy4_b	5.32	97.07	92.89	54.06	90.54	92.70	0.50	6.80
hy4_c	3.98	96.86	92.34	54.22	89.55	93.60	0.40	6.00
hy4_in_a	4.56	96.88	92.38	54.01	90.24	93.40	0.50	6.10
hy4_in_b	3.99	96.85	92.33	54.21	89.07	93.30	0.50	6.20
hy4_in_c	4.10	97.10	92.82	53.92	89.13	91.90	0.60	7.50
Cg_a	4.58	97.08	92.78	57.20	90.07	77.90	0.70	21.40
Cg_b	5.33	97.19	92.98	57.15	90.08	75.80	0.70	23.50
Cg_c	4.62	96.74	92.10	57.32	89.01	77.10	0.60	22.30
Cg_in_a	4.36	96.97	92.54	57.12	89.89	79.70	0.40	19.90
Cg_in_b	4.99	96.94	92.48	57.08	89.67	78.90	0.50	20.60
Cg_in_c	4.94	96.80	92.22	57.16	89.2	79.70	0.60	19.70

hy4_^*^ and Cg_^*^ represent the control groups of hy4 and Cg01; hy4_in_^*^ and Cg_in_^*^ represent the induced groups of hy4 and Cg01 (“^*^” means “a” or “b” or “c” in Table). Each experiment was performed in triplicate.

**Figure 5:**
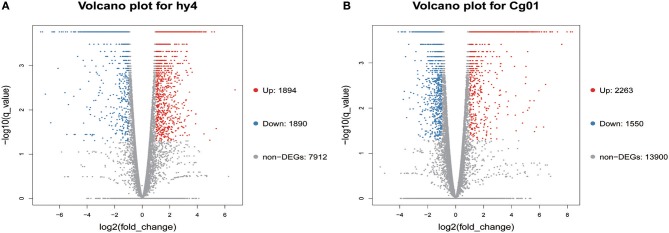
Volcano plots of differentially expressed genes in two endophytic fungi **(A)** *P. polonicum* hy4; **(B)** *C. gloeosporioides* Cg01. Volcano plots visualize the DEGs between the two different groups. *Q*-values ≤ 0.05 and | log2(fold_change) |≥1 were used as a threshold to determine the significance of DEGs. Red dots: up-regulated genes; Blue dots: down-regulated genes; Gray dots: genes with no significant difference.

Distinctive expression patterns of genes between the control and induced group in hy4 and Cg01 provide an opportunity to find important genes that were functionally responsive to the addition of *H. serrata* extracts and might correlate with HupA biosynthesis. Among the 11,696 genes in hy4, 3,784 (32.4%) were differentially expressed after induction [*Q*-value ≤ 0.05, |log2(fold_change) |≥ 1], with 1,894 (16.2%) up-regulated (Figure 5; Table S2). In Cg01, 3,813 genes (21.53%) were differentially expressed (*Q*-value ≤ 0.05, |log2(fold_change) | ≥ 1), with 2,263 (19.0%) up-regulated (Figure 5; Table S3).

**Figure 6:**
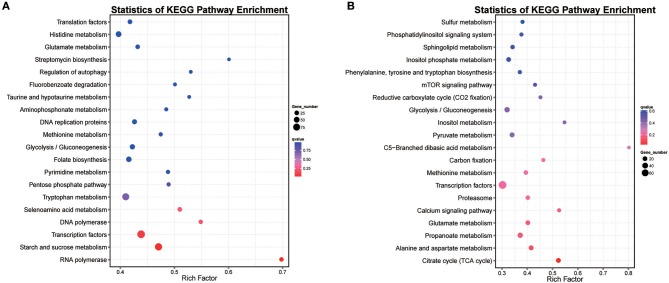
Scatter plot of top 20 enriched KEGG pathways for genes with significant expression levels in two endophytic fungi **(A)** *P. polonicum* hy4 **(B)** *C. gloeosporioides* Cg01. A high *Q*-value is represented by blue and a low *Q*-value is represented by red. Bubble size indicates DEG number (increases with DEG number).

DEGs in hy4 and Cg01 belonged to different functional pathways, including transporters (160/144), transcription factors (93/79), tryptophan metabolism (78/63), etc (Tables S4, S5). However, there was no shared pathway between hy4 and Cg01 with *Q*-value ≤ 0.05. KEGG enrichment pathways were “RNA polymerase” and “Starch and sucrose metabolism” in hy4, while for Cg01 it was “Citrate cycle”. With *P*-value ≤ 0.05, the shared enrichment pathways “Glycolysis/Gluconeogenesis” and “Transcription factors” were most affected by the addition of *H. serrata* extracts. Because HupA-producing ability was improved after induction in both hy4 and Cg01, the shared enrichment pathways with similar expression pattern were the focuses. With *Q*-value ≤ 1, the shared enrichment pathways with similar expression pattern were “Glycolysis/Gluconeogenesis,” “Tryptophan metabolism,” “Methionine metabolism,” and “Glutamate metabolism” (Figure 6).

**Figure 7:**
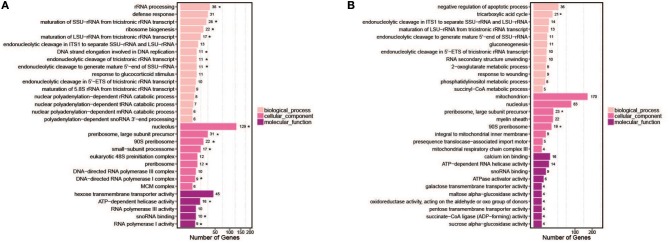
Bar chart of top 30 GO terms for genes with significantly enhanced expression in two endophytic fungi **(A)** *P. polonicum* hy4; **(B)** *C. gloeosporioides* Cg01. Symbol ^*^ indicates enrichment was significant (*Q*-value < 0.05). The number after the GO term is the number of genes in the enrichment GO terms.

The GO consortium provides a standardized and hierarchical vocabulary (GO terms) to describe the function of gene products and classifies genes into functional categories. Of the significantly changed genes, 30 significantly enriched GO terms were obtained, divided into three main categories (biological processes, cellular components, and molecular functions) (Figure 7). The most enriched GO pathways (*Q*-value ≤ 0.05) included 16 pathways in hy4 and 3 in Cg01 (Figure 7; Tables S6, S7). The shared enrichment pathways between hy4 and Cg01 were preribosome, large subunit precursor, and 90S preribosome.

**Figure 8:**
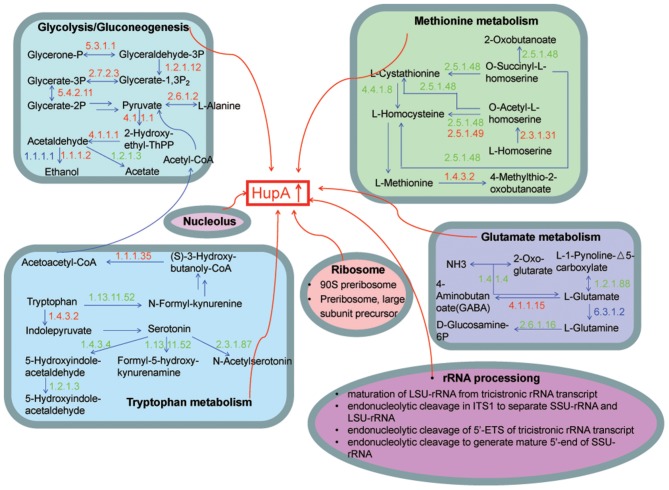
Model illustrating the KEGG pathways and GO function clusters of the potential DEGs involved in HupA biosynthesis. The number above each horizontal arrow is the EC number of the enzyme catalyzing this reaction, while red represents upregulation, green represents downregulation, and blue represents genes that both up- and down-regulate.

We included for comprehensive consideration the DEGs in GO and KEGG pathways with similar expression pattern in hy4 and Cg01. The DEGs were mostly involved in carbon metabolism (Glycolysis/Gluconeogenesis), nitrogen metabolism (Methionine metabolism, Tryptophan metabolism, Glutamate metabolism) and in the nucleolus, ribosome, and rRNA processing (Figure 8). Most of the shared DEGs in “Glycolysis/Gluconeogenesis” were upregulated, while most shared DEGs in amino acid metabolism (Methionine metabolism, Tryptophan metabolism, Glutamate metabolism) were downregulated (Figure 8).

### Identification and Expression of the Genes in the Proposed HupA Biosynthesis Pathway

Because the genes LDC, CAO, PKS, BBE, P450, and 2OGD had been proposed to participate in the HupA biosynthetic pathway in plants [5], we examined these genes and their expression in the two endophytic fungi. These included 1 LDC gene, 6 CAO genes, 27 PKS genes, 2 BBE genes, 111 P450s, and 35 2OGDs in hy4, while Cg01 included 2 LDC genes, 12 CAO genes, 47 PKS genes, 4 BBE genes, 280 P450s, and 39 2OGDs (Tables S8–S10). Both hy4 and Cg01 are capable of producing HupA, indicating hy4 and Cg01 may have shared HupA-biosynthesis genes, which are orthologous in these two fungi or clustered together in the phylogenetic analysis. Therefore, we have done orthologous analysis (Table S11) as well as phylogenetic analysis between hy4 and Cg01 (Figures S2, S3).

Within the LDC genes, *PpLDC, CgLDC1*, and *CgLDC2* were orthologues (Table S8). The sole LDC gene (PpLDC) in hy4 was down-regulated. The homologous *CgLDC1* was up-regulated while *CgLDC2* was down-regulated.

There were 7 CAO genes in Cg01 which had orthologous genes in hy4 (Table S8). In addition, in the phylogenetic tree of CAO, *CgCAO5* clustered with *PpCAO1* and *Shiraia* sp. slf14 (Figure S2), which has been reported to produce HupA (Zhu et al., 2010). In these 8 CAO genes, 4 were downregulated in the induced group and 3 showed low expression (NOTEST, Table S8). The expression of the remaining one (*CgCAO4*) was also low with no significance difference between the two groups (FPKM = 0.6~0.9). For the orthologues of *CgCAO4* in hy4, one was downregulated and one did not show a significantly different expression between the two groups (Table S8). Considering that CAO was proposed as the prophase skeleton synthase in HupA, the induced HupA increase might be more closely related to later modification. Therefore, *CgCAO4* was considered the candidate biosynthesis gene.

There were 27 and 47 PKS genes (including PKS-NRPS hybrids) in hy4 and Cg01, respectively (Table S8). Almost all PKSs in the two endophytic fungi were type I, except *CgPKS40* which was type III. Though there were more PKS genes in Cg01 than in hy4, most of the PKS genes in Cg01 (including hybrid, 37/47) were seldom expressed (NOTEST), and three were down-regulated, one up-regulated, and the others were not differentially expressed (Table S8). PKSs with indispensable domains and orthologues between hy4 and Cg01 were the center of our focus. From the known chemical structure of HupA, PKS that biosynthesize HupA should only contain AT (acyl transferase), KS (ketosynthase), ACP (acyl carrier protein), and/or TE (thioesterase) domains. Only 3 PKS had these indispensable domains in hy4 and Cg01 (Table S8). These genes were either NOTEST or down-regulated in the DEG analysis in both fungi (Table S8). A total of 15 gene pairs were orthologues, including one pair clustering together in the phylogenetic tree (Table S8, Figure S3) between hy4 and Cg01, all of which were NOTEST or down-regulated in either fungus. Therefore, no candidate PKS genes were obtained for further validation by these two methods. We thus selected the upregulated *CgPKS35* and relatively highly expressed PKS genes *CgPKS14* and *CgPKS21* as the candidates for validation.

All BBE genes in hy4 showed low expression (NOTEST, Table S8), while in Cg01 3 were downregulated and 1 showed low expression (NOTEST, Table S8). Thus, we proposed that these identified BBE do not likely participate in HupA biosynthesis.

We identified 111 P450 genes in hy4 and 280 in Cg01. In both endophytic fungi, the superfamily with the most genes was CYP65, with 109 and 203 genes in hy4 and Cg01, respectively (Table S9). A total of 35 and 39 2OGD sequences were detected by searching for the 2OG-FeII_Oxy motif against the whole genomes of hy4 and Cg01, respectively (Table S10). As most biosynthetic genes for SMs were clustered together, we identified the genomic locations of putative P450 and 2OGD genes to explore gene clustering. We regarded 2 P450s, 2 2OGDs, one P450, and one 2OGD located within 10 genes as a gene cluster. There were 15 and 43 P450 gene clusters in hy4 and Cg01, respectively, with hy4_CYP_09247-RA and hy4_CYP_09249-RA up-regulated, and no P450 cluster upregulated in Cg01. The 2OGD gene clusters were fewer, with 3 and 4 in hy4 and Cg01, respectively. However, no 2OGD gene clusters were upregulated after induction. In the gene clusters of containing both P450 and 2OGD genes, there was no cluster with all genes upregulated. Most showed low expression (NOTEST), or one upregulated while others showed no detectable difference in expression.

Taken together, we listed the LDC genes *CgLDC1* and *CgLDC2*, the CAO gene *CgCAO4*, and the PKS genes *CgPKS35, CgPKS14*, and *CgPKS21* as candidate HupA biosynthesis genes for validation.

### Identification and Expression Analysis of Epigenetics Related Genes

Epigenetic modifiers, of both histone methylation and acetylation, have played important roles in the regulation of secondary metabolism (Williams et al., 2008). Nine HMTs, Eighteen histone acetyltransferases (HATs), and Thirteen HDACs were identified in each endophyte, across hy4 and Cg01 (Table S12). Among the HATs, 12 GNATs (Gcn5-related N-acetyltransferases) were predicted in hy4 and Cg01. HDACs include conventional (Zn-dependent Class I, II, and IV protein lysine deacetylases) and NAD^+^-dependent deacetylases. In both fungi, 6 conventional and 7 NAD^+^-dependent HDACs were identified.

The expression of HMT, HAT, and HDAC orthologues between hy4 and Cg01 was analyzed in each fungus. In HMT orthologous groups, only *PpClr4*-*CgClr4* were expressed and showed a similar pattern—both were downregulated after induction (Table S12). For HATs, only *PpSpt10-1*-*CgSpt10* were downregulated. In another HAT orthologous group *PpSAS-2*-*CgSAS-2, CgSAS-2* was downregulated with *q*-value ≤ 0.05, while *PpSAS-2* was downregulated with *p*-value ≤ 0.05. Among the HDAC DEGs, there was no HDAC orthologous group expressed with a similar pattern. In the groups of *PpClr3*- *CgClr3, PpSir2-7*- *CgSir2-3*, and *PpSir2-6*- Cg*Sir2-6*, the changes in HDAC expression in hy4 were opposite to those in Cg01. Because both deletion and overexpression of HDAC could result in SM accumulation with the same tendency (Studt et al., 2013), the HDAC DEGs with opposite patterns in hy4 and Cg01 were listed as candidate genes (*CgClr3, CgSir2-3, CgSir2-6*).

### Functional Analysis of Candidate Genes in HupA Biosynthesis

Gene deletion was conducted to analyze the association between the candidate genes and HupA biosynthesis. LDC genes (*CgLDC1, CgLDC2*), CAO genes (*CgCAO4*), PKS genes (*CgPKS35, CgPKS14, CgPKS21*) and epigenetic genes (*CgClr4, CgSpt10, CgSAS-2, CgClr3, CgSir2-3, CgSir2-6*) were knocked out by homologous deletion. All genes were deleted successfully except *CgSpt10* and *CgSir2-3* (Figures S4–S9).

**Figure 9:**
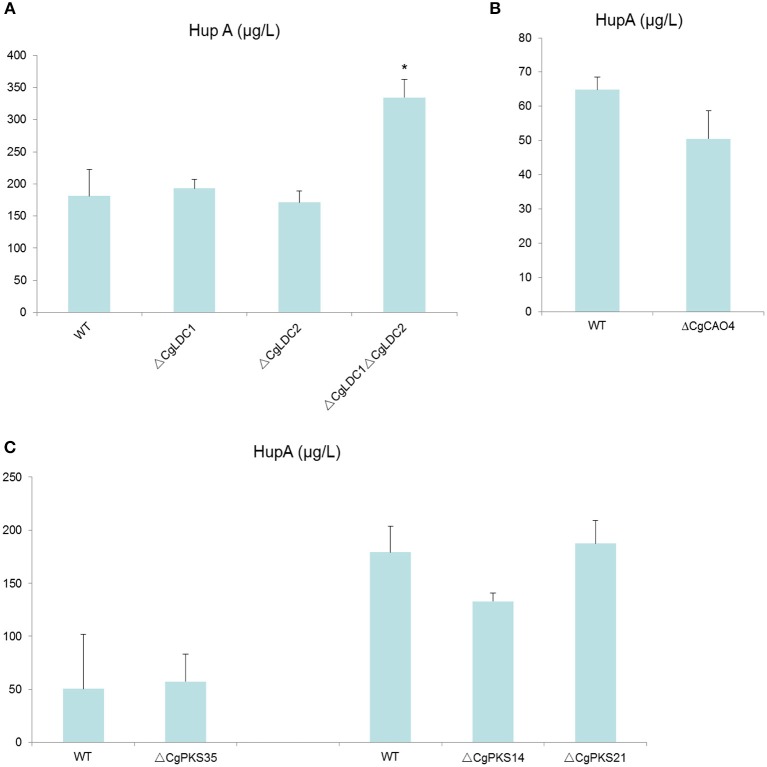
Comparison of HupA production from culture extracts from wild type (WT) *C. gloeosporioides* Cg01 and LDC, CAO, and PKS gene knock out mutants (**A**: LDC; **B**: CAO; **C**: PKS). The experiment was performed in triplicate. Mean values and standard deviations are given. Asterisks above the bars denote significant differences in the measurements of the indicated strains compared to the WT. ^*^p < 0.05.

To eliminate the compensation effect, we not only knocked-out *CgLDC1* and *CgLDC2* individually, but also knocked-out both at one time. However, we found that HupA production of the single deletion mutants of LDC was not changed, and that of double deletion of LDC genes increased by 83% (Figure 9; Figure S10). For the CAO and PKS genes, there was no differential HupA production in the single deletion mutant fermentation broth (Figure 9; Figures S11, S12).

**Figure 10:**
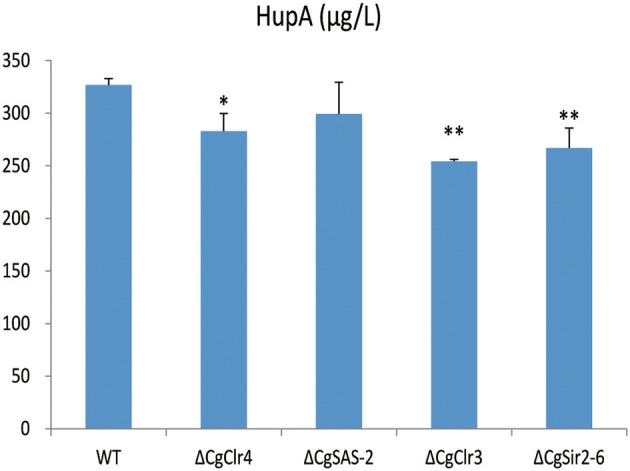
Comparison of HupA production from culture extracts from wild type (WT) *C. gloeosporioides* Cg01 and Δ*CgClr4*, Δ*CgSAS-2*, Δ*CgClr3*, and Δ*CgSir2-6* mutants. The experiment was performed in triplicate. Mean values and standard deviations are given. Asterisks above the bars denote significant differences in the measurements of the indicated strains compared to the WT. ^*^p < 0.05; ^**^p < 0.01.

**Figure 11:**
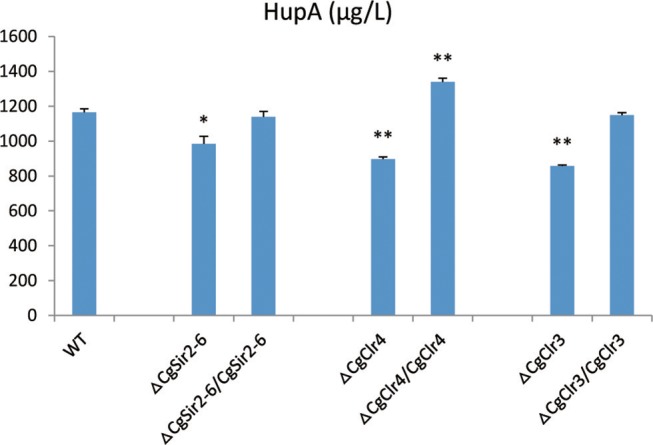
Comparison of HupA production from culture extracts from wild type (WT) *C. gloeosporioides* Cg01, epigenetic gene knock-out mutants, and the corresponding retro-complementation strains. The experiment was performed in triplicate. Mean values and standard deviations are given. Asterisks above the bars denote significant differences in the measurements of the indicated strains compared to the WT. ^*^p < 0.05; ^**^p < 0.01.

The HupA production of deletion mutant strains Δ*CgClr4*, Δ*CgClr3*, and Δ*CgSir2-6* were reduced by 13.46, 22.26, and 18.37%, respectively, while the mutant strain Δ*CgSAS-2* was not changed (Figure 10; Figure S13). Retro-complementation of the *CgClr4, CgClr3*, and *CgSir2-6* mutants restored the total HupA production (Figure 11; Figure S14).

## Discussion

HupA, a natural acetylcholinesterase inhibitor for treatment of Alzheimer's disease, is the SM of both Huperziaceae plants and endophytic fungi. After induction by the extracts of *H. serrata*, HupA production of hy4 and Cg01 increased. Relevant DEGs were mostly clustered in carbon and nitrogen metabolism and in nucleolus, ribosome, and rRNA processing. It is reported that primary carbon and nitrogen metabolism could influence secondary metabolism (Drew and Demain, 1977). Generally, the presence of rapidly utilized carbon and nitrogen sources inhibits secondary metabolism (Drew and Demain, 1977). Some SMs are produced when the rapidly used carbon and nitrogen in the medium are exhausted (Ward and Packter, 1974; Kennel, 1977). In this study, most upregulated genes were in the DEGs enrichment KEGG pathway “Glycolysis/Gluconeogenesis.” Therefore, the rapid use of glucose may be of benefit for the biosynthesis of the SM HupA. In addition, the action of primary metabolism on secondary metabolism might be the result of precursor activity or an even more important role, i.e., an inducer for biosynthesis of the SMs (Drew and Demain, 1977). One example involves tryptophan as a stimulatory precursor of ergoline alkaloid in *Claviceps* (Robbers and Floss, 1970; Vining, 1970). In the DEGs enrichment KEGG pathway, most genes involved in nitrogen metabolism were downregulated. It is possible that the nitrogen metabolites were the precursors or inducers for HupA biosynthesis, so it is repressed in the amino acid metabolism pathways “Methionine metabolism,” “Tryptophan metabolism,” and “Glutamate metabolism.”

Ribosomes, serving as the site of biological protein synthesis, are important cellular organelles that can respond to nutrition level and regulate growth rate (Gutteridge et al., 2010). Ribosomes also play a pivotal role in SM biosynthesis by regulating relevant gene expression. Therefore, the rate of ribosome processing could influence the protein synthesis ability and the synthesis of SMs (Ochi et al., 2004). “Ribosome engineering,” proposed by Ochi, could activate or enhance the production of SMs by targeting S12, RNA polymerase, and other ribosomal proteins and translation factors (Ochi, 2017). In this study, the addition of the inducing extracts of *H. serrata* may act on ribosomes or RNA polymerase, which results in a similar ribosomal stress mechanism, and consequently synthesis of the SM HupA.

The biosynthetic pathway of HupA was ambiguous not only in plants but also in fungi. Most of the proposed biosynthetic genes in endophytic fungi were only characterized and expressed *in vitro*, such as LDC, CAO, and PKS genes (Sun et al., 2012; Yang et al., 2016; Xu et al., 2017). Previous studies could not establish the direct relationship between these genes and HupA biosynthesis. Zhang et al. over-expressed LDC and CAO genes in the endophytic fungus *C. gloeosporioides* ES026 and some of the over-expressed transformants produced higher HupA than the wild type (Zhang et al., 2015). However, there is no repeat in the HupA analysis of these transformants fermentation. In the current study, there was no significant effect on HupA production in the mutant when the single LDC genes, CAO genes, and PKS genes of *C. gloeosporioides* Cg01 were knocked-out. However, when we knocked-out the *CgLDC1* and *CgLDC2* genes simultaneously, HupA production increased significantly. In *P. polonicum* hy4, the expression of the LDC gene *PpLDC*, the sole LDC gene discovered, decreased after induction. In addition, we found an interesting phenomenon in analyzing the expression of homologous genes between *P. polonicum* hy4 and *C. gloeosporioides* Cg01. For a pair of homologous genes that encoded lysine biosynthetic regulatory proteins or the fungal specific transcription factor (hy4_05232-RA—Cg01_07009-RA), expression was down-regulated in both *P. polonicum* hy4 and *C. gloeosporioides* Cg01 after induction. We suspect that HupA synthesized in endophytic fungi is likely different from the mechanism in plants. HupA and lysine may be end products of a branched biosynthetic pathway, with some shared pathway or precursors, like penicillin formation in *Aspergillus nidulans* (Busch et al., 2003). Moreover, a recent paper reported on the biosynthesis of pipecolic acid (Pip) of *H. serrata*, a non-protein amino acid containing a piperidine ring, which is the biosynthesis precursor of piperidine alkaloids (Xu et al., 2018). This report demonstrated *in vitro* that lysine aminotransferase HsAld1 can transfer amino groups from L-lysine to pyruvate. The intermediate 1,2-dehydropipecolic acid (1,2-DP) then isomerized to 2,3-dehydropipecolic acid (2,3-DP) and was reduced to Pip by lysine cyclization deamination enzyme *HsSard4*. Therefore, it is most likely that HupA could be biosynthesized via other pathways.

The origins of the SMs in endophytes have been the source of debate. Some investigators suggested the source of fungal SMs as the host plant due to inconsistent and unsustainable production over several generations (Venugopalan and Srivastava, 2015). However the discovery of the SM-synthesizing fungi isolated from non-SM producing plants indicates that fungi could be the source (Flores-Bustamante et al., 2010). We also found that the endophytes isolated from non-HupA producing plants could produce HupA with extracts of *H. serrata*, contradicting the idea of fungal SM being a host plant adduct. Regarding the SM-biosynthesis pathway in endophytes, some studies suggest that HGT may have taken place between plants and endophytes during their long period of association, while the others hypothesize that plants and endophytes may have co-evolved parallel pathways to produce SM (Venugopalan and Srivastava, 2015). Most of the identified fungal Taxol-biosynthesis genes were found to be homologous to their plant counterparts (with >96% sequence similarity), which strongly supports the HGT theory (Staniek et al., 2009; Zhang et al., 2009). However, the sheer complexity of the Taxol biosynthetic pathway, which involves more than 20 steps localized to different subcellular compartments, with genes possibly scattered over different plant chromosomes, has raised doubts over the possibility of transfer of the entire pathway from the host plant to the endophytes. The case of gibberellin biosynthetic pathways which show several differences in fungi and higher plants might support the co-evolution theory rather than HGT theory (Hedden et al., 2001; Tudzynski, 2005). Using our methods of homology and DEG analysis with the knock-out validation, it was difficult to find the genuine HupA-biosynthesis gene in fungi. Therefore, we supposed that the HupA-biosynthesis genes in fungi might co-evolve with the plant rather than being acquired through HGT.

The epigenetic regulation of chromatin is a complex process and certain histone modifications can play dual roles as they can have different effects depending on the physical location of the chromatin stretch. For example, in *Saccharomyces cerevisiae*, H3K4 tri-methylation is a permissive mark for gene transcription in euchromatic regions but is also required for gene silencing at mating type loci and sub-telomeric regions (Bryk et al., 2002; Mueller et al., 2006). Deletion of *cclA*, a yeast *Bre2* ortholog involved in H3K4 methylation, activated the expression of cryptic SM clusters in *A. nidulans*. The deletion strain produced several additional metabolites, among them monodictyphenone (MDP), emodin, and four emodin derivatives not previously observed in *A. nidulans* (Bok et al., 2009). In general, histone deacetylation is associated with gene silencing (Bannister and Kouzarides, 2011). However, there is increasing evidence that HDACs are also required for gene activation (Shwab et al., 2007; Lee et al., 2009; Li et al., 2011). Deletion of the *A. fumigatus* HDAC *hdaA* gene increased the production of several SMs but decreased production of gliotoxin whereas over-expression *hdaA* increased production of gliotoxin (Lee et al., 2009). In this study, the expression of HDAC gene *CgSir2-6* was upregulated after induction. When it was knocked-out, HupA production of the mutants decreased as expected. However, expression of the HMT gene *CgClr4* and the HDAC gene *CgClr3* was down-regulated in DEG analysis. Surprisingly, deletion of *CgClr4* and *CgClr3* did not result in the increased accumulation of HupA but in a decrease. A similar effect was observed for *FfHda1* in *Fusarium fujikuroi* and *cphos2* in *Claviceps purpurea*: both deletion and overexpression of the HDAC genes led to the same accumulation of SMs (Studt et al., 2013). These results are representative of the complexity of the epigenetic regulation in SM biosynthesis. HDAC deacetylates not only the histone but also transcription factor, where deacetylation is closely related with recruit HAT. When HDACs were knocked out, the transcription factor could not be deacetylated, and consequently could not recruit HAT to acetylate the histone located adjacent to the HupA biosynthesis gene, leading to the decoupling of pathway-specific transcription factor and biosynthesis genes (Studt et al., 2013). When HDACs were downregulated, the histone adjacent to the HupA biosynthesis gene was acetylated, and HupA production increased. In addition, HDAC *Sir2* and *Clr3* are reported to be required for *Clr4* activity with telomeric or centromeric heterochromatin assembly (Alper et al., 2013). In regulation of HupA biosynthesis, whether *CgClr4, CgClr3*, and *CgSir2-6* function together remains unknown.

## Conclusion

The results of DEG analysis and functional validation demonstrate that the HupA biosynthetic pathway in endophytic fungus *C. gloeosporioides* Cg01 might be different from that proposed in plants. The HupA-biosynthesis genes in endophytic fungi might co-evolve with the plant machinery rather than being acquired through HGT. The HMT genes (*CgClr4*) and HDAC genes (*CgClr3, CgSir2-6*) associated with histone modification were involved in the regulation of HupA biosynthesis. This is the first report on epigenetic modification analysis in high value SM-producing endophytic fungi. These findings shed new light on HupA biosynthesis and regulation by HupA-producing endophytic fungi, and it is crucial for larger production of HupA from fungi.

## Author Contributions

DL conceived this study. XK analyzed the data, drafted the manuscript, and participated in the experiments. CL analyzed the genomes and differentially expressed genes. PS and LH participated in the data analysis and experiments. RL and JL assembled the genomes. XX and BX participated in the experiment design. All authors have read and approved the final manuscript.

### Conflict of Interest Statement

The authors declare that the research was conducted in the absence of any commercial or financial relationships that could be construed as a potential conflict of interest.

## Supplementary Material

Figure S1 HPLC profiles of culture extracts from *P. polonicum* hy4 and *C. gloeosporioides* Cg01. a: HupA; b: PDB+extracts; c: *P. polonicum* hy4; d: *P. polonicum* hy4+extracts; e: *C. gloeosporioides*; f: *C. gloeosporioides*+ extracts.

Figure S2 Maximum likelihood phylogeny for CAOs in *P. polonicum* hy4, *C. gloeosporioides* Cg01, and other CAOs deposited in NCBI.

Figure S3 Maximum likelihood phylogeny for PKSs in *P. polonicum* hy4 and *C. gloeosporioides* Cg01. The domain structures of PKSs were predicted by antiSMASH and the KS domains were used for phylogenetic analysis. MEGA 7.0 was used to determine the best model (LG) and construct the phylogenetic tree.

Figure S4 PCR amplification verified *CgLDC1* ***(A)****, CgLDC2* ***(B)*** deletion in WT *C. gloeosporioides* and *CgLDC1* deletion in the *CgLDC2* gene knockout mutant **(C)**. **(A/C-a)** target fragment (580 bp); (A/C-b) 5′-flanking of target gene + HPT gene (3,256 bp); **(A/C-c)** 3′-flanking of target gene + HPT gene (3,060 bp). M: **(A-a)** 1 kb plus DNA ladder **(A-b) (A-c) (C)** 2 kb plus DNA ladder (TransGen Biotech). **(B-a)** target fragment (598 bp); **(B-b)** 5′-flanking of target gene + HPT gene (3488 bp); **(B-c)** 3′-flanking of target gene + HPT gene (3,337 bp). M: 1kb Plus DNA marker (TransGen Biotech).

Figure S5 PCR amplification verifed *CgCAO4* deletion in WT *C. gloeosporioides*. **(A)** target fragment (1,190 bp); **(B)** 5′-flanking of target gene + HPT gene (3,159 bp); **(C)** 3′-flanking of target gene + HPT gene (3,023 bp). 1–14: transformants; M: **(A)** 2kb Plus, DNA marker **(B), (C)**1 kb plus DNA ladder (TransGen Biotech).

Figure S6 PCR amplification verifed *CgPKS14* **(A)***, CgPKS21* **(B)***, CgPKS35* **(C)** deletion in WT *C. gloeosporioides*. **(A-a)** target fragment (1,200 bp); **(A-b)** 5′-flanking of target gene + HPT gene (3,646 bp); **(A-c)** 3′-flanking of target gene + HPT gene (2,959 bp). M **(A)**: 2kb Plus DNA marker (TransGen Biotech). **(B-a)** target fragment (1,101 bp); **(B-b)** 5′-flanking of target gene + HPT gene (3040 bp); **(B-c)** 3′-flanking of target gene + HPT gene (2,708 bp). M: **(B-a)** 2kb Plus DNA marker **(B-b/c)**1 kb plus DNA ladder (TransGen Biotech). **(C-a)** target fragment (1,001 bp); **(C-b)** 5′-flanking of target gene + HPT gene (3,206 bp); **(C-c)** 3′-flanking of target gene + HPT gene (2,927 bp). M: **(C-a)** 2kb Plus DNA marker **(C-b/c)** 1 kb plus DNA ladder (TransGen Biotech).

Figure S7 PCR amplification verifed *CgClr4* deletion in WT *C. gloeosporioides*. **(A)** target fragment (449 bp); **(B)** 5′-flanking of target gene + HPT gene (3,466 bp); **(C)** 3′-flanking of target gene + HPT gene (2,940 bp). 1–14: transformants; M: 1 kb plus DNA ladder (TransGen Biotech).

Figure S8 PCR amplification verifed *CgSAS-2* deletion in WT *C. gloeosporioides*. **(A)** target fragment (942 bp); **(B)** 5′-flanking of target gene + HPT gene (2,923 bp); **(C)** 3′-flanking of target gene + HPT gene (3,086 bp). 1–14: transformants; M: 1 kb plus DNA ladder (TransGen Biotech).

Figure S9 PCR amplification verifed *CgClr3, CgSir2-6* deletion in WT *C. gloeosporioides*. **(A-a)** target fragment (616 bp); **(A-b)** 5′-flanking of target gene + HPT gene (2,905 bp); **(A-c)** 3′-flanking of target gene + HPT gene (3,019 bp). **(B-a)** target fragment (770 bp); **(B-b)** 5′-flanking of target gene + HPT (3,777 bp); **(B-c)** 3′-flanking of target gene + HPT gene (2,213 bp). M: 1 kb plus DNA ladder (TransGen Biotech).

Figure S10 HPLC profiles of culture extracts from WT *C. gloeosporioides* Cg01 and LDC gene-knockout mutants **(A**: Δ*CgLDC2*, **B**: Δ*CgLDC1*, **C**: Δ*CgLDC1*Δ*CgLDC2***)**. a: HupA; b: HupB; c: PDB +extracts; d: WT; e: WT +extracts; f: mutants; g: mutants +extracts.

Figure S11 HPLC profiles of culture extracts from WT *C. gloeosporioides* Cg01 and Δ*CgCAO4* mutant. a: HupA; b: HupB; c: PDB+extracts; d: WT; e: WT +extracts; f: Δ*CgCAO4*; g: Δ*CgCAO4*+extracts.

Figure S12 HPLC profiles of culture extracts from WT *C. gloeosporioides* Cg01 and PKS gene knock-out mutants **(A**: Δ*CgPKS35*; **B**: Δ*CgPKS21*; **C**: Δ*CgPKS14***)**. a: HupA; b: HupB; c: PDB +extracts; d: WT; e: WT +extracts; f: mutant; g: mutant +extracts.

Figure S13 HPLC profiles of culture extracts from WT *C. gloeosporioides* Cg01 and histone lysine modification gene knock-out mutants **(A**: Δ*CgClr4*; **B**: Δ*CgSAS-2*; **C**: Δ*CgClr3*; **D**: Δ*CgSir2-6***)**. a: HupA; b: PDB +extracts; c: WT; d: WT +extracts; e: mutants; f: mutants +extracts.

Figure S14 HPLC profiles of culture extracts from WT *C. gloeosporioides* Cg01, histone lysine modification gene knock-out mutants, and the retro-complementation strains **(A**: Δ*CgClr4*; **B**: Δ*CgClr3*; **C**: Δ*CgSir2-6***)**. a: HupA; b: PDB +extracts; c: WT +extracts; d: mutants +extracts; e: complementation strains + extracts.

Table S1 Gene-knockout and retro-complementation primers.

Table S2 Gene annotation and expression of *P. polonicum* hy4.

Table S3 Gene annotation and expression of *C. gloeosporioides* Cg01.

Table S4 KEGG pathway enrichment analysis of the differentially expressed genes between the induced group and the control group in *P. polonicum* hy4.

Table S5 KEGG pathway enrichment analysis of the differentially expressed genes between the induced group and the control group in *C. gloeosporioides* Cg01.

Table S6 GO pathway enrichment analysis of the differentially expressed genes between the induced group and the control group in *P. polonicum* hy4.

Table S7 GO pathway enrichment analysis of the differentially expressed genes between the induced group and the control group in *C. gloeosporioides* Cg01.

Table S8 Expression of the LDC, CAO, BBE and PKS genes in *C. gloeosporioides* Cg01 and *P. polonicum* hy4.

Table S9 Annotation of P450s in *P. polonicum* hy4 and *C. gloeosporioides* Cg01.

Table S10 Gene expression of 2OGD in *P. polonicum* hy4 and *C. gloeosporioides* Cg01.

Table S11 Orthologues between *P. polonicum* hy4 and *C. gloeosporioides* Cg01.

Table S12 Gene expression of histone modification genes in *P. polonicum* hy4 and *C. gloeosporioides* Cg01.

## Abbreviations

PCGs: protein-coding genes
ML: maximum likelihood
DEGs: differentially expressed genes
LDC: lysine decarboxylase
CAO: copper amine oxidase
PKS: polyketides synthases
BBE: berberine bridge enzyme
2OGD: 2-oxoglutarate-dependent dioxygenases
HGT: horizontal gene transfer
HAT: histone acetyltransferase
HMT: histone methyltransferase
HDAC: histone deacetylase
NCBI RefSeq: National Center of Biotechnology Information Reference Sequence
UniProtKB: UniProt Knowledgebase
GO: Gene ontology
KEGG: Kyoto Encyclopedia of Genes and Genomes
KOG: EuKaryotic Orthologous Groups
HPLC: High Performance Liquid Chromatography.
