# Supplementary figures and images for: Genomic Characterization Provides New Insights Into the Biosynthesis of the Secondary Metabolite Huperzine a in the Endophyte Colletotrichum gloeosporioides Cg01
Source: Front Microbiol. 2019 Jan 8;9:3237. doi: 10.3389/fmicb.2018.03237 (PMC6331491; doi:10.3389/fmicb.2018.03237)

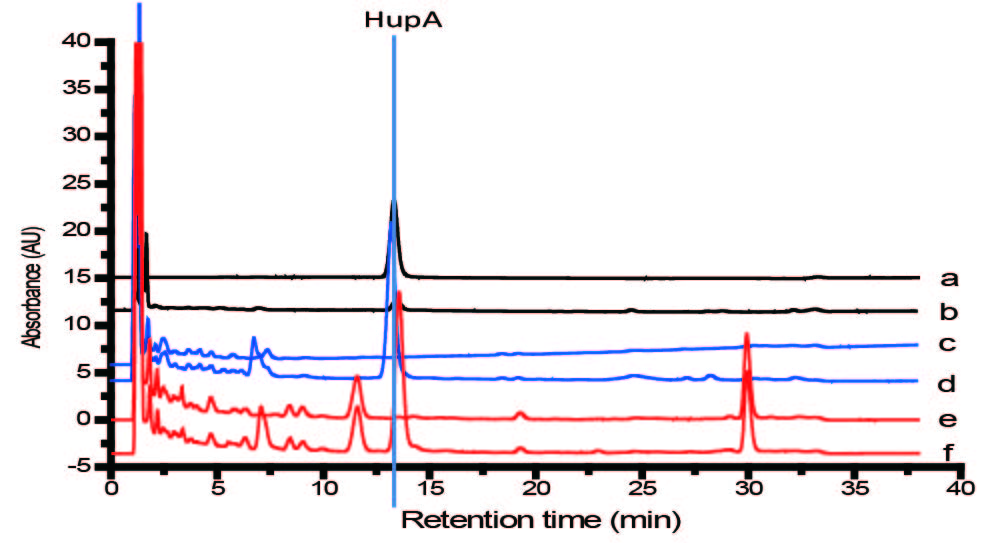

Supplement: Figure S1 — HPLC profiles of culture extracts from P. polonicum hy4 and C. gloeosporioides Cg01. a: HupA; b: PDB+extracts; c: P. polonicum hy4; d: P. polonicum hy4+extracts; e: C. gloeosporioides; f: C. gloeosporioides+ extracts. [file Image_1.JPEG]

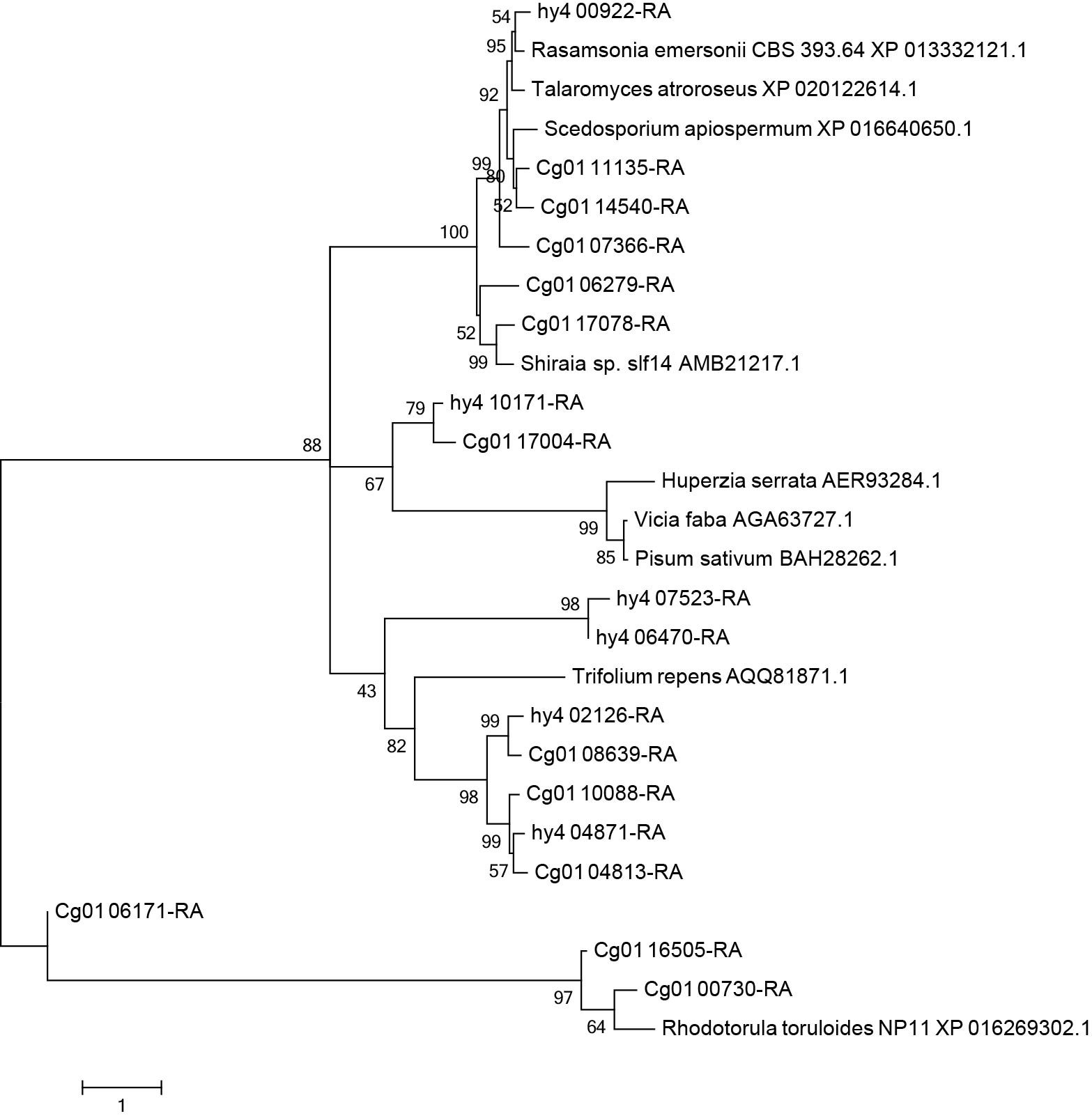

Supplement: Figure S2 — Maximum likelihood phylogeny for CAOs in P. polonicum hy4, C. gloeosporioides Cg01, and other CAOs deposited in NCBI. [file Image_2.JPEG]

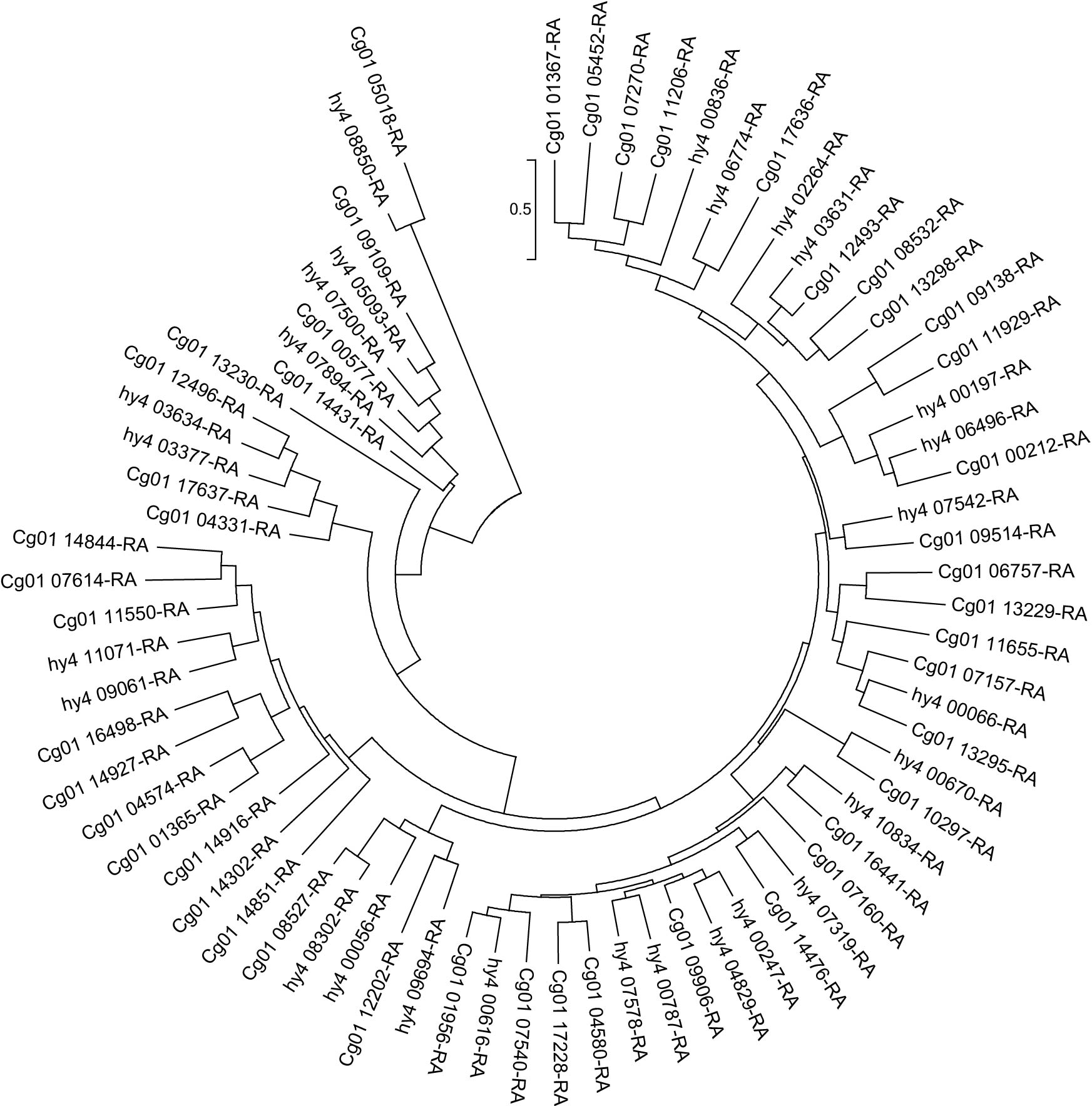

Supplement: Figure S3 — Maximum likelihood phylogeny for PKSs in P. polonicum hy4 and C. gloeosporioides Cg01. The domain structures of PKSs were predicted by antiSMASH and the KS domains were used for phylogenetic analysis. MEGA 7.0 was used to determine the best model (LG) and construct the phylogenetic tree. [file Image_3.JPEG]

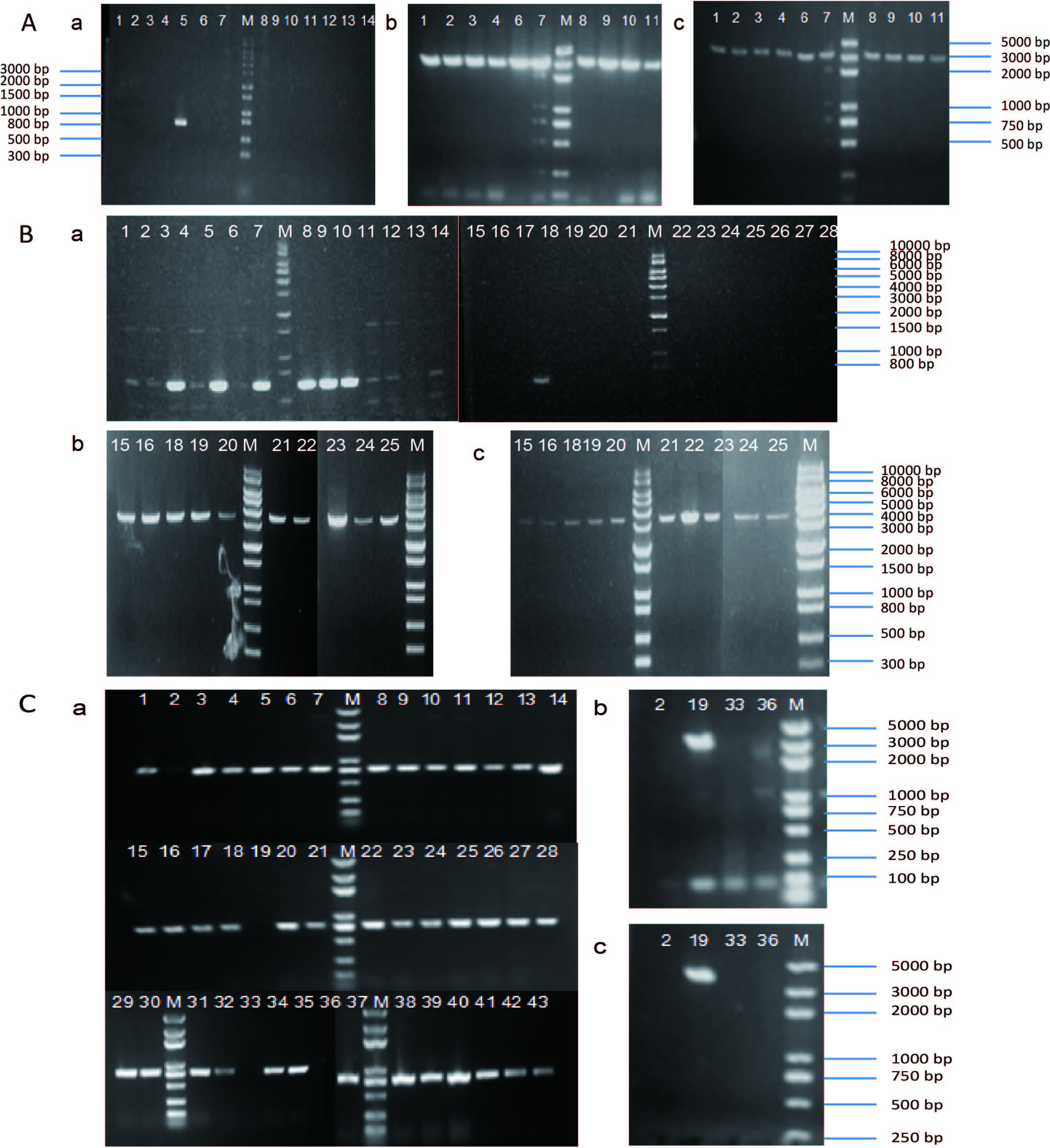

Supplement: Figure S4 — PCR amplification verified CgLDC1 (A), CgLDC2 (B) deletion in WT C. gloeosporioides and CgLDC1 deletion in the CgLDC2 gene knockout mutant (C). (A/C-a) target fragment (580 bp); (A/C-b) 5′-flanking of target gene + HPT gene (3,256 bp); (A/C-c) 3′-flanking of target gene + HPT gene (3,060 bp). M: (A-a) 1 kb plus DNA ladder (A-b) (A-c) (C) 2 kb plus DNA ladder (TransGen Biotech). (B-a) target fragment (598 bp); (B-b) 5′-flanking of target gene + HPT gene (3488 bp); (B-c) 3′-flanking of target gene + HPT gene (3,337 bp). M: 1kb Plus DNA marker (TransGen Biotech). [file Image_4.JPEG]

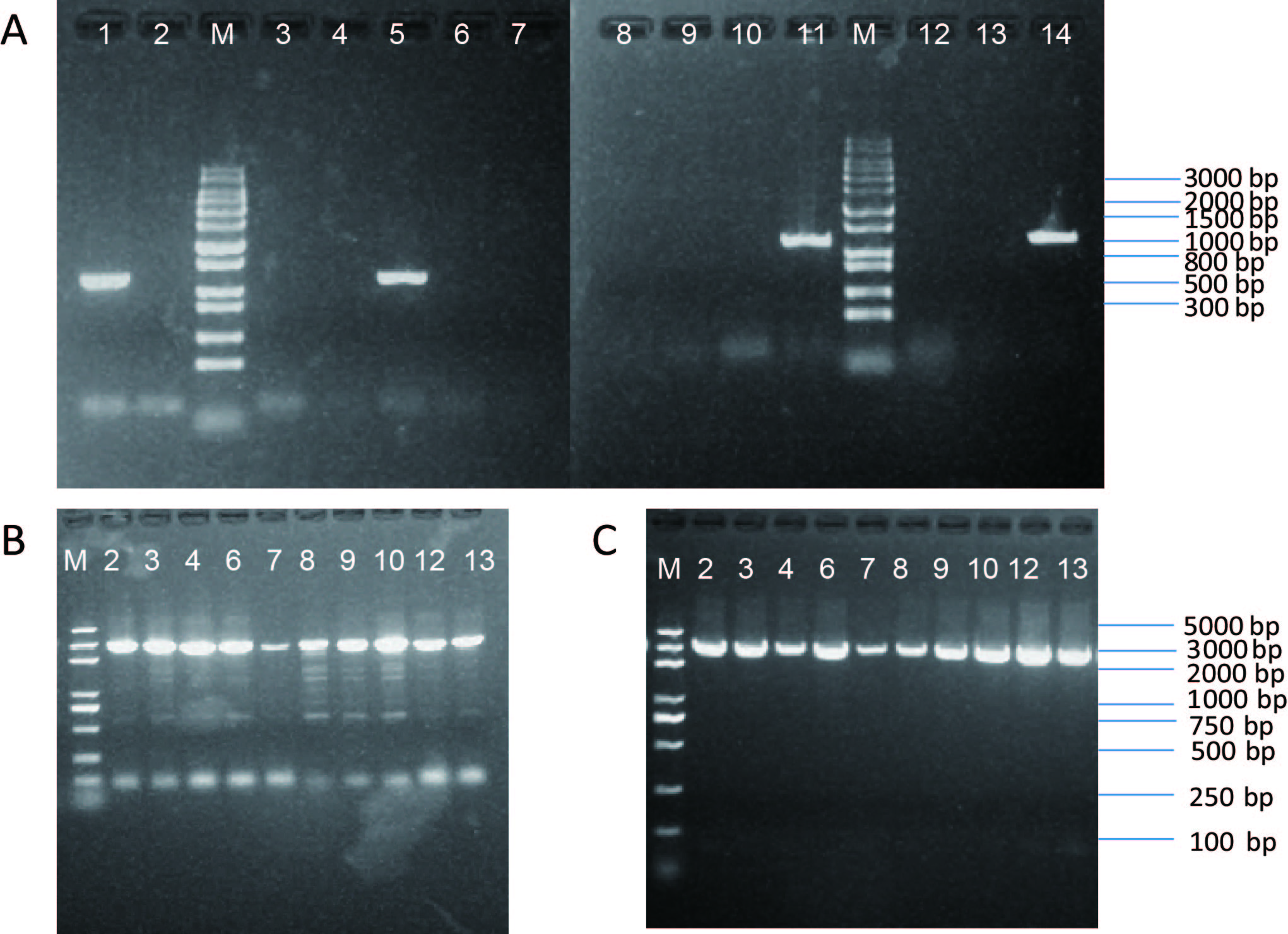

Supplement: Figure S5 — PCR amplification verifed CgCAO4 deletion in WT C. gloeosporioides. (A) target fragment (1,190 bp); (B) 5′-flanking of target gene + HPT gene (3,159 bp); (C) 3′-flanking of target gene + HPT gene (3,023 bp). 1–14: transformants; M: (A) 2kb Plus, DNA marker (B), (C)1 kb plus DNA ladder (TransGen Biotech). [file Image_5.JPEG]

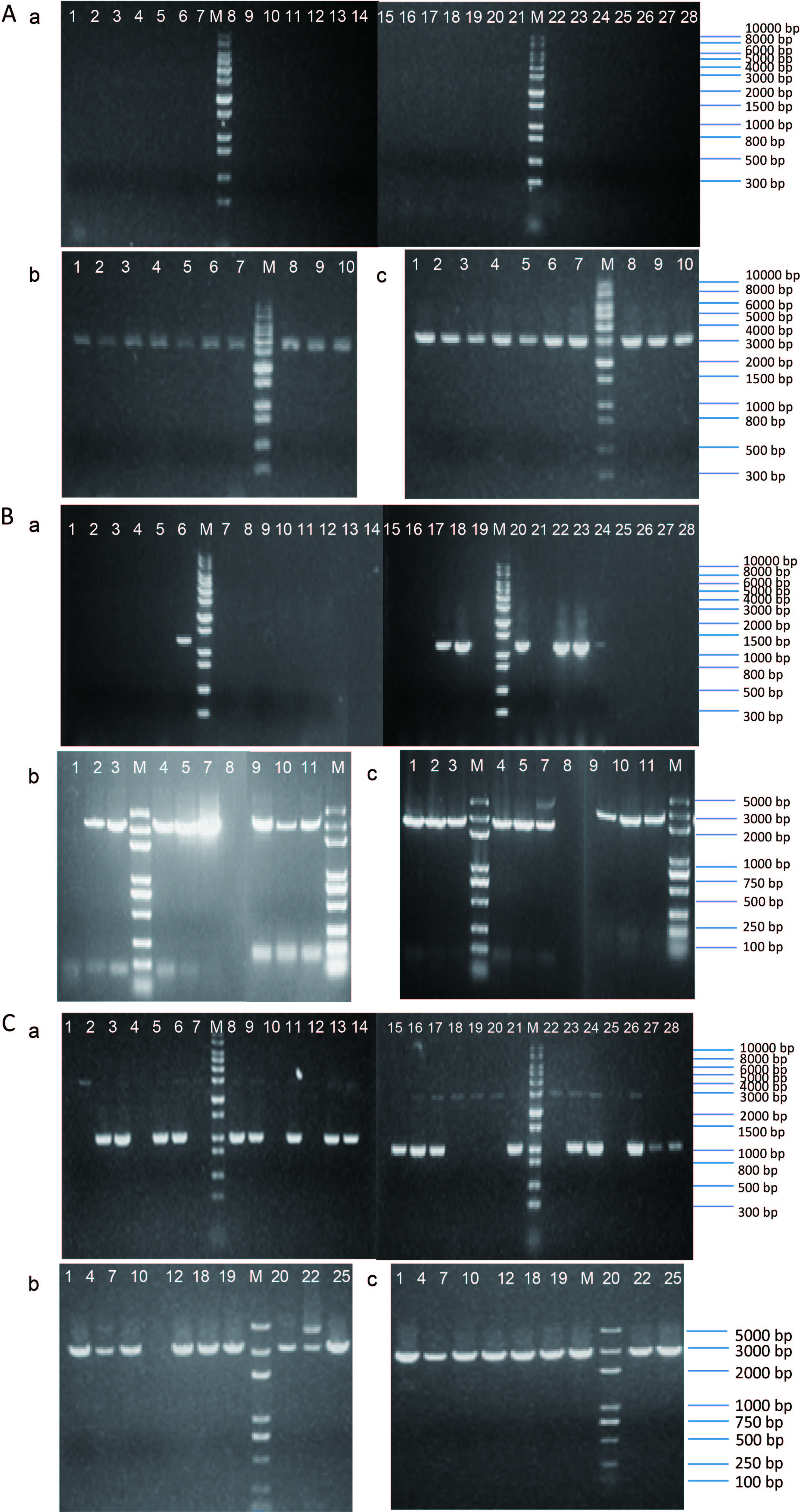

Supplement: Figure S6 — PCR amplification verifed CgPKS14 (A), CgPKS21 (B), CgPKS35 (C) deletion in WT C. gloeosporioides. (A-a) target fragment (1,200 bp); (A-b) 5′-flanking of target gene + HPT gene (3,646 bp); (A-c) 3′-flanking of target gene + HPT gene (2,959 bp). M (A): 2kb Plus DNA marker (TransGen Biotech). (B-a) target fragment (1,101 bp); (B-b) 5′-flanking of target gene + HPT gene (3040 bp); (B-c) 3′-flanking of target gene + HPT gene (2,708 bp). M: (B-a) 2kb Plus DNA marker (B-b/c)1 kb plus DNA ladder (TransGen Biotech). (C-a) target fragment (1,001 bp); (C-b) 5′-flanking of target gene + HPT gene (3,206 bp); (C-c) 3′-flanking of target gene + HPT gene (2,927 bp). M: (C-a) 2kb Plus DNA marker (C-b/c) 1 kb plus DNA ladder (TransGen Biotech). [file Image_6.JPEG]

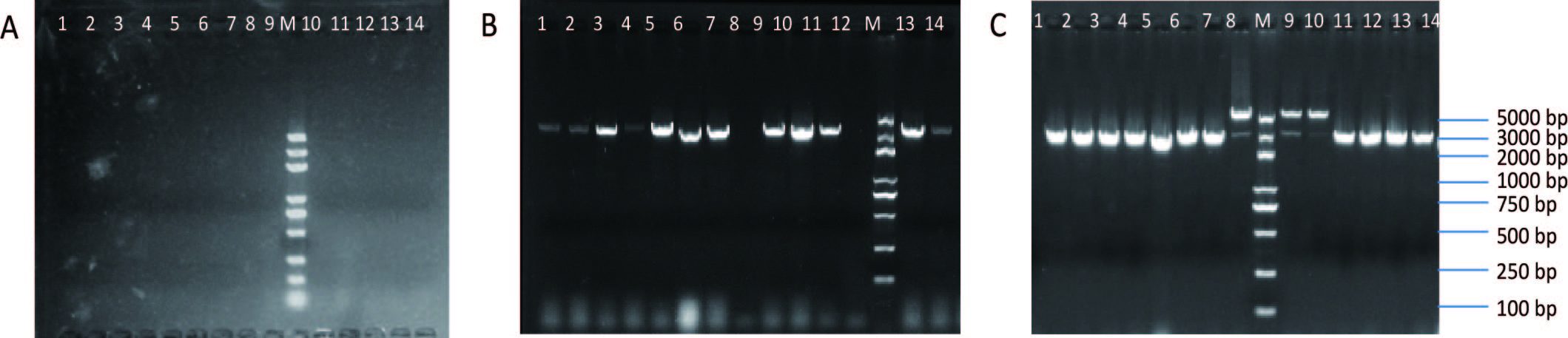

Supplement: Figure S7 — PCR amplification verifed CgClr4 deletion in WT C. gloeosporioides. (A) target fragment (449 bp); (B) 5′-flanking of target gene + HPT gene (3,466 bp); (C) 3′-flanking of target gene + HPT gene (2,940 bp). 1–14: transformants; M: 1 kb plus DNA ladder (TransGen Biotech). [file Image_7.JPEG]

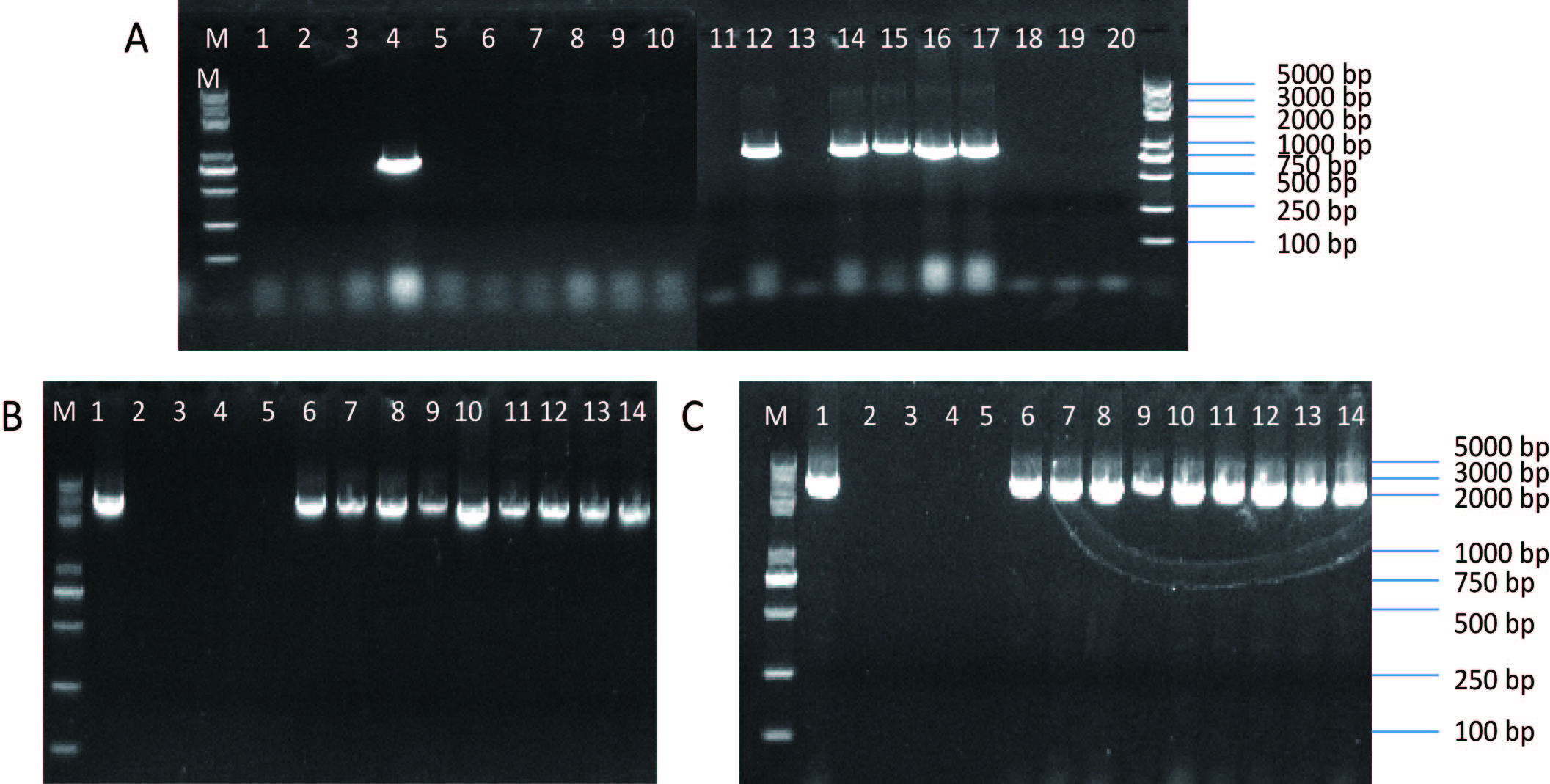

Supplement: Figure S8 — PCR amplification verifed CgSAS-2 deletion in WT C. gloeosporioides. (A) target fragment (942 bp); (B) 5′-flanking of target gene + HPT gene (2,923 bp); (C) 3′-flanking of target gene + HPT gene (3,086 bp). 1–14: transformants; M: 1 kb plus DNA ladder (TransGen Biotech). [file Image_8.JPEG]

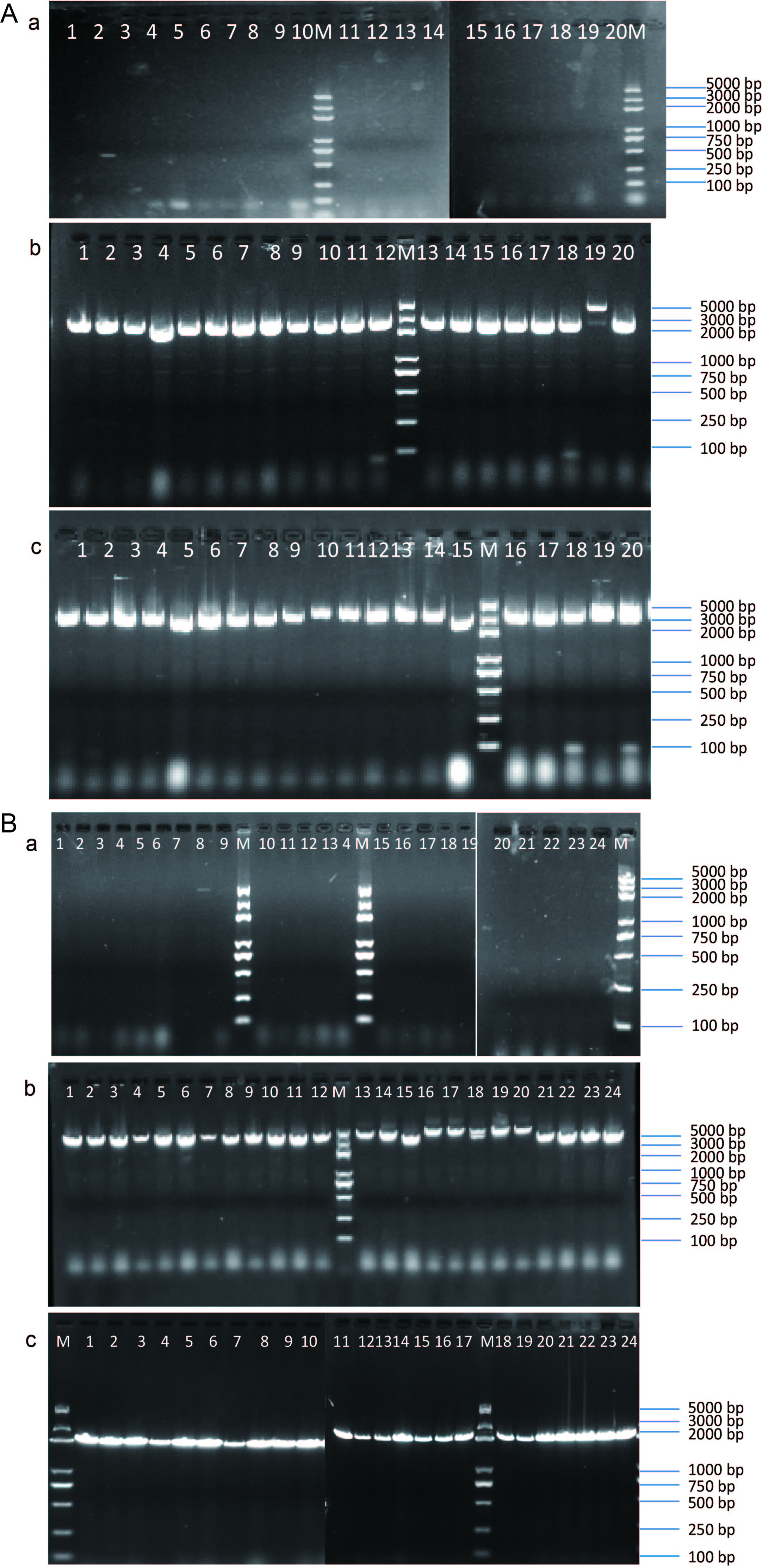

Supplement: Figure S9 — PCR amplification verifed CgClr3, CgSir2-6 deletion in WT C. gloeosporioides. (A-a) target fragment (616 bp); (A-b) 5′-flanking of target gene + HPT gene (2,905 bp); (A-c) 3′-flanking of target gene + HPT gene (3,019 bp). (B-a) target fragment (770 bp); (B-b) 5′-flanking of target gene + HPT (3,777 bp); (B-c) 3′-flanking of target gene + HPT gene (2,213 bp). M: 1 kb plus DNA ladder (TransGen Biotech). [file Image_9.JPEG]

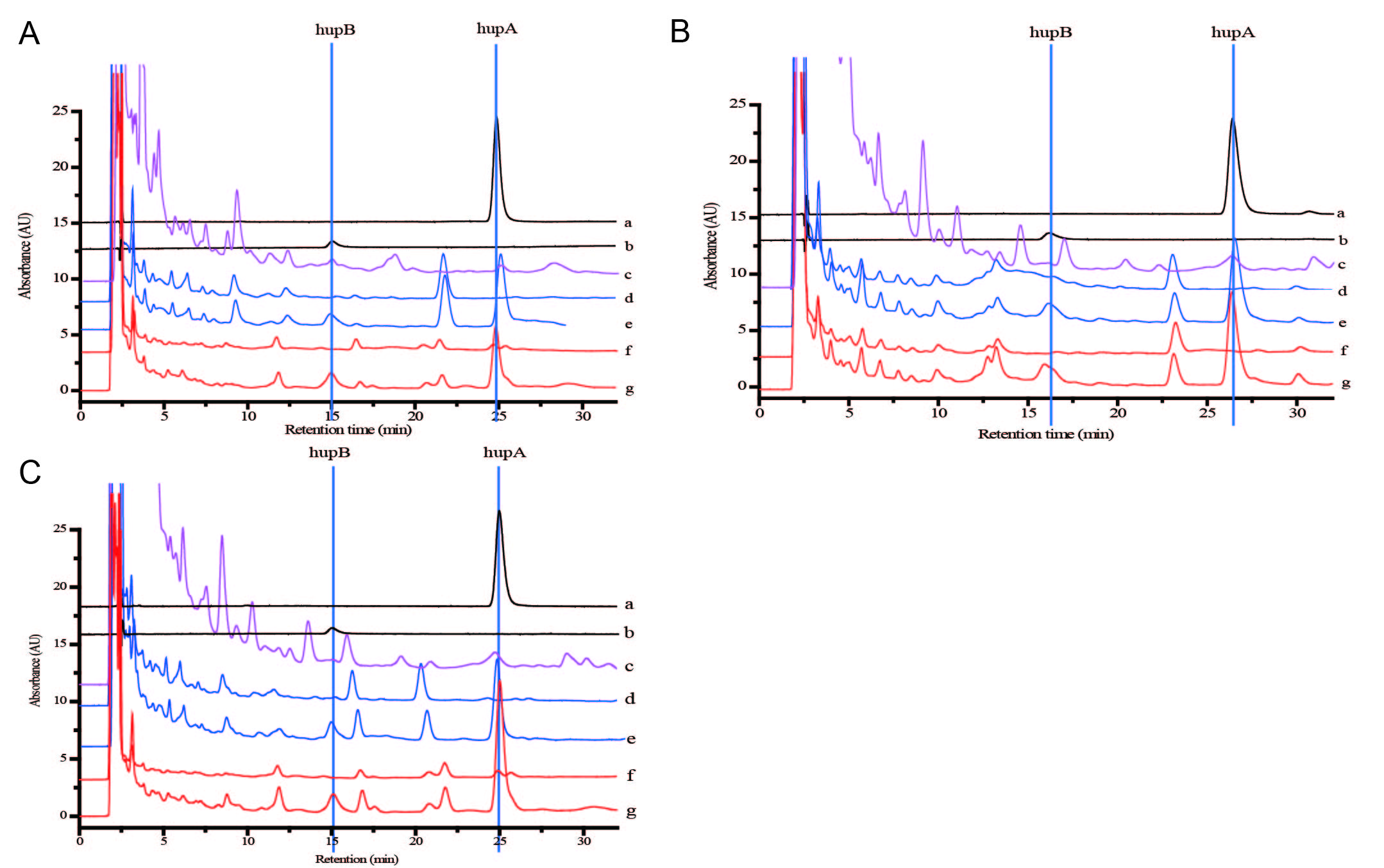

Supplement: Figure S10 — HPLC profiles of culture extracts from WT C. gloeosporioides Cg01 and LDC gene-knockout mutants (A: ΔCgLDC2, B: ΔCgLDC1, C: ΔCgLDC1ΔCgLDC2). a: HupA; b: HupB; c: PDB +extracts; d: WT; e: WT +extracts; f: mutants; g: mutants +extracts. [file Image_10.JPEG]

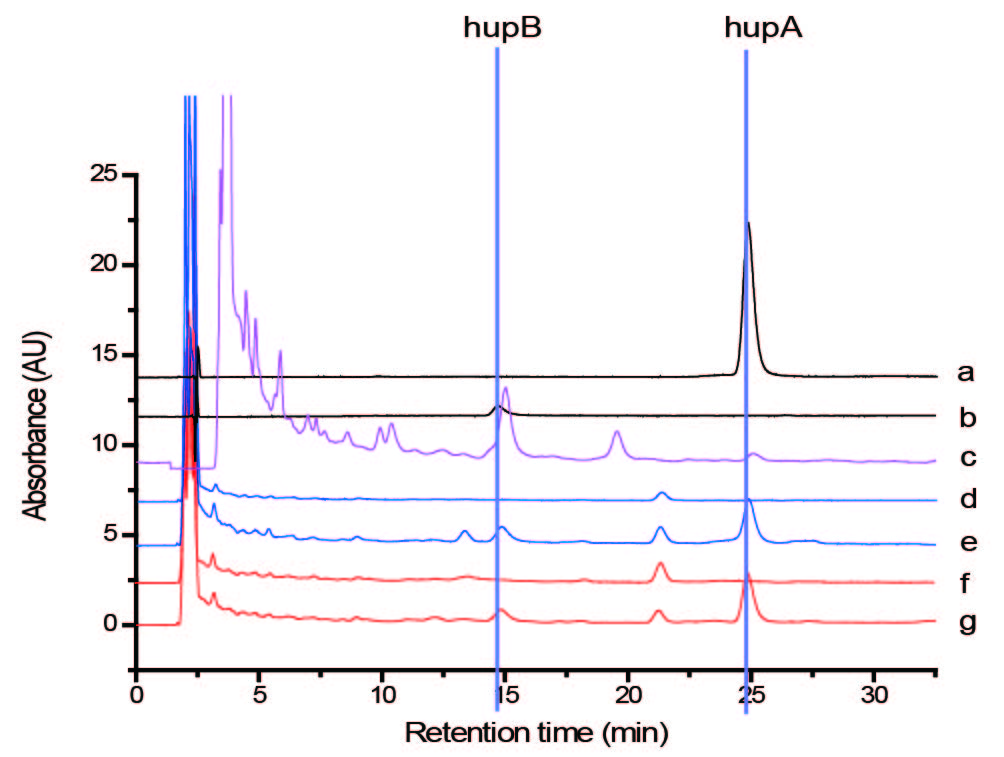

Supplement: Figure S11 — HPLC profiles of culture extracts from WT C. gloeosporioides Cg01 and ΔCgCAO4 mutant. a: HupA; b: HupB; c: PDB+extracts; d: WT; e: WT +extracts; f: ΔCgCAO4; g: ΔCgCAO4+extracts. [file Image_11.JPEG]

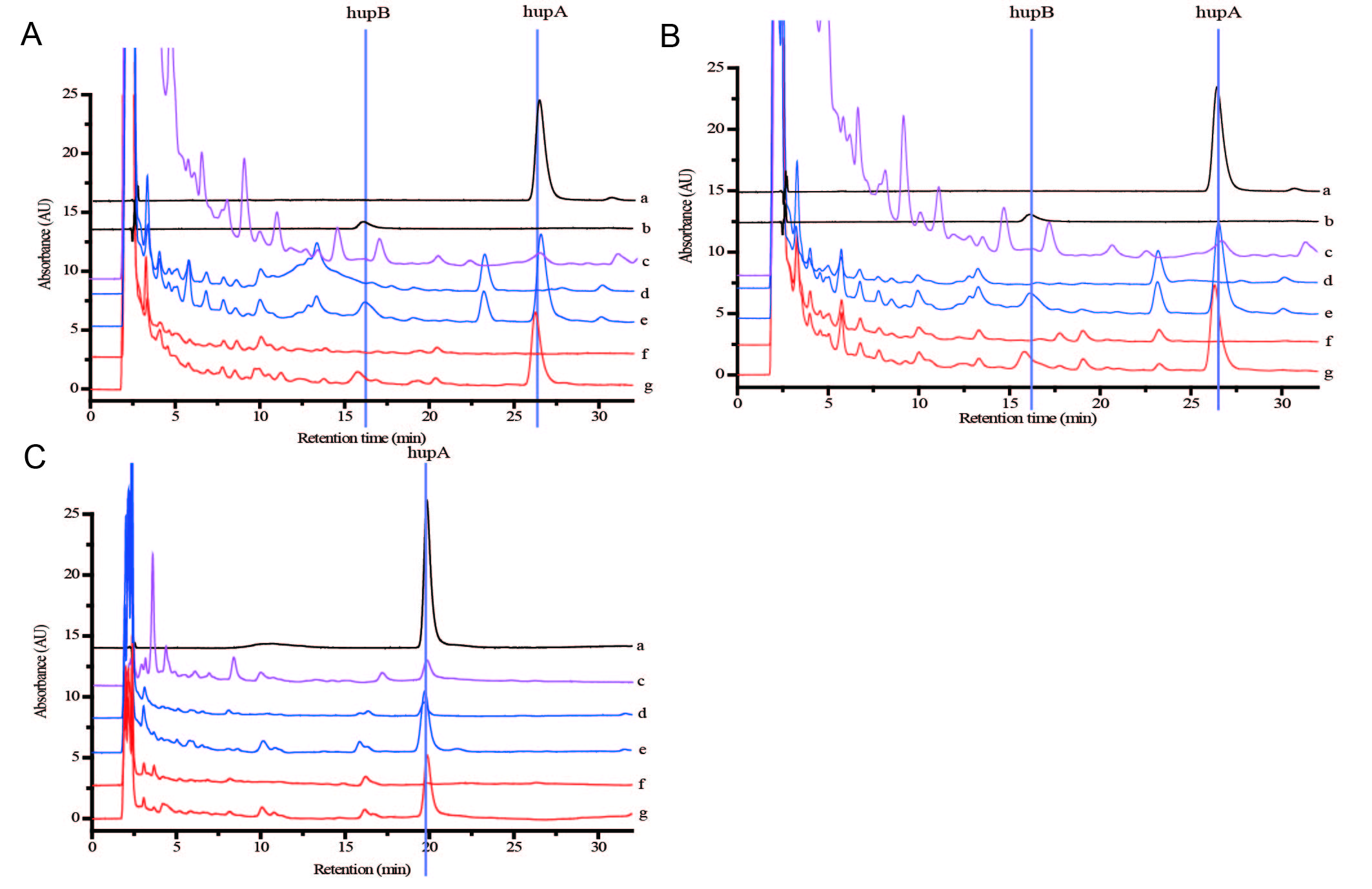

Supplement: Figure S12 — HPLC profiles of culture extracts from WT C. gloeosporioides Cg01 and PKS gene knock-out mutants (A: ΔCgPKS35; B: ΔCgPKS21; C: ΔCgPKS14). a: HupA; b: HupB; c: PDB +extracts; d: WT; e: WT +extracts; f: mutant; g: mutant +extracts. [file Image_12.JPEG]

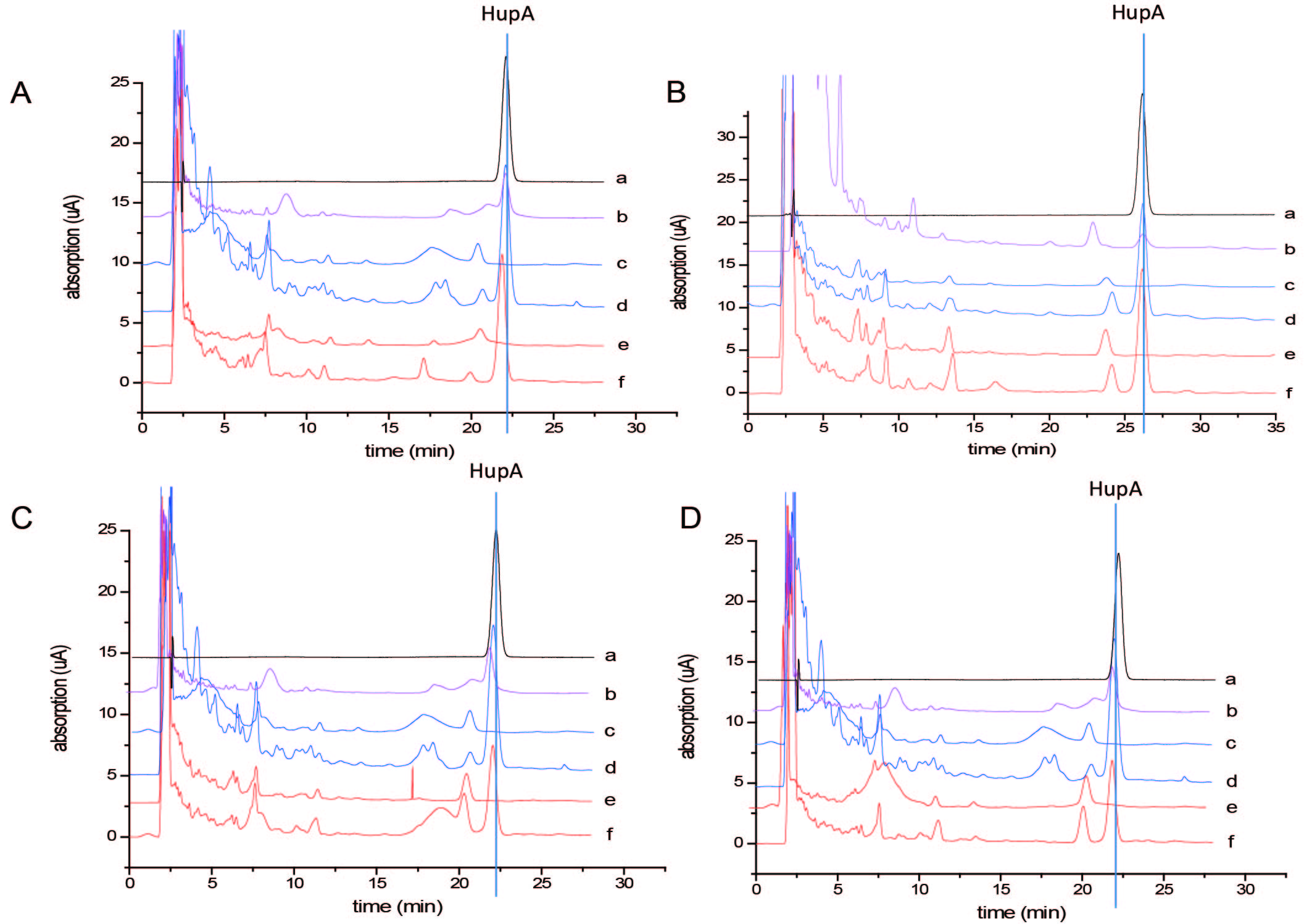

Supplement: Figure S13 — HPLC profiles of culture extracts from WT C. gloeosporioides Cg01 and histone lysine modification gene knock-out mutants (A: ΔCgClr4; B: ΔCgSAS-2; C: ΔCgClr3; D: ΔCgSir2-6). a: HupA; b: PDB +extracts; c: WT; d: WT +extracts; e: mutants; f: mutants +extracts. [file Image_13.JPEG]

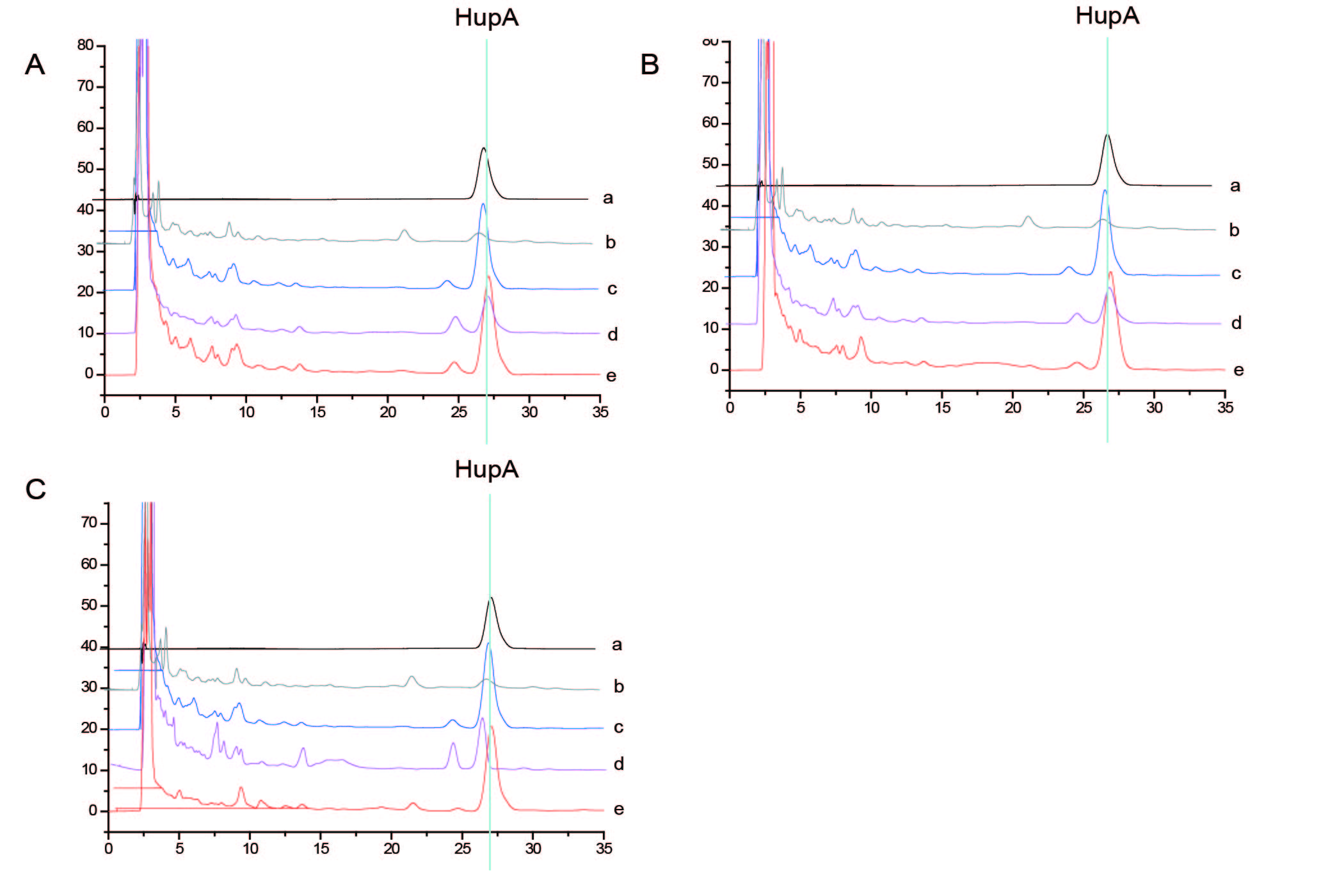

Supplement: Figure S14 — HPLC profiles of culture extracts from WT C. gloeosporioides Cg01, histone lysine modification gene knock-out mutants, and the retro-complementation strains (A: ΔCgClr4; B: ΔCgClr3; C: ΔCgSir2-6). a: HupA; b: PDB +extracts; c: WT +extracts; d: mutants +extracts; e: complementation strains + extracts. [file Image_14.JPEG]
